# Predicting the Carbon Footprint of Building Material Using Machine Learning: Production Phase, Secondary Material Use, and Recycling Potential

**DOI:** 10.3390/ma19214666

**Published:** 2026-11-02

**Authors:** Karol Skiba, Aleksandra Mach, Joanna Sagan, Roman Kinasz

**Affiliations:** 1 AGH Doctoral School, AGH University of Krakow, Al, Mickiewicza 30, 30-059 Krakow, Poland; 2 Faculty of Civil Engineering and Resource Management, AGH University of Krakow, Al, Mickiewicza 30, 30-059 Krakow, Poland; amach@agh.edu.pl (A.M.); joanna.sagan@agh.edu.pl (J.S.); rkinash@agh.edu.pl (R.K.)

**Keywords:** machine learning, ML, life cycle assessment, LCA, environmental product declarations, EPD, carbon footprint, global warming potential, building materials, secondary materials, recycling potential, module D

## Abstract

**Highlights:**

- Machine learning models predict production-stage GWP (A1–A3) from EPD indicators with test R^2^ = 0.96 and a relative MAE of 13%.
- Non-renewable primary energy use (PENRT) accounts for more than half of total SHAP predictor importance.
- Module D GWP is harder to predict (test R^2^ = 0.79) and generalizes poorly to unseen manufacturers and product categories.
- Secondary materials (SM) gain importance in module D prediction, but their effect is not statistically conclusive.
- The analysis covered 2214 ÖKOBAUDAT records (EN 15804+A2) after IQR-based outlier removal; robustness was tested with alternative outlier handling and with nested and grouped cross-validation.

**Abstract:**

Environmental product declarations (EPDs) provide carbon footprint data for construction products. This study evaluated machine learning predictions of global warming potential (GWP–total) for the production stage (A1–A3) and module D from EPD resource-use and waste indicators, focusing on secondary material use (SM). After interquartile range removal of target outliers, the analysis used 2214 A1–A3 records and 1765 EPDs with module D data from ÖKOBAUDAT. Extreme Gradient Boosting (XGBoost), Random Forest, and Support Vector Regression (SVR) were tuned by 5-fold cross-validation; predictor importance was assessed with Shapley Additive Explanations values and ablation. XGBoost achieved the highest test coefficient of determination (R^2^) 0.963 for A1–A3, 0.791 for module D, with relative mean absolute errors of 13% and 27%, respectively. Its advantage over Random Forest was mostly within bootstrap uncertainty. Total use of non-renewable primary energy resources (PENRT) dominated A1–A3 predictions, whereas radioactive waste disposed (RWD), total use of renewable primary energy resources (PERT), and SM gained importance for module D. Removing SM changed module D R^2^ by −0.03 (95% confidence interval: −0.07 to 0.00). Grouped validation reduced module D R^2^ to approximately 0.4 for unseen groups. The models may support plausibility checks and estimation of missing module D values.

## 1. Introduction

The 2024/2025 Global Status Report for Buildings and Construction refers approximately 34% of global CO_2_ emissions to the buildings and construction sector [1]. As operational energy efficiency improves, embodied greenhouse gas emissions account for a larger share of building life-cycle impacts [2]. Circular economy (CE) strategies seek to reduce demand for raw material resources through reuse, recycling, and the incorporation of secondary materials into construction products [3,4]. Life-cycle assessment (LCA) provides a framework for evaluating potential environmental impacts across the stages included within a product system. For construction products, the EN 15804:2012+A2:2019 [5] standard specifies core rules for reporting LCA-based environmental indicators, including total global warming potential (GWP–total). In this context, GWP–total, expressed in kg CO_2_-eq, is used as the quantitative indicator of the carbon footprint associated with the analysed life-cycle stage. Throughout this paper, the term GWP refers to GWP–total unless otherwise stated.

Environmental product declarations (EPDs) communicate these indicators in a standardised format [5]. Ning et al. [6] analyzed more than 16,000 EPDs from nine EPD libraries and found that the building and construction sector had the greatest data availability in their sample. They also identified limited reporting beyond the production stage, particularly for the use phase. ÖKOBAUDAT provides EPD-derived environmental indicators alongside generic LCA datasets across a wide range of construction-product categories [7]. However, a common database does not ensure direct comparability: methodological choices can produce different EPD results for the same product [8].

Recovered and recycled constituents are increasingly investigated as alternatives to conventional raw materials in construction [9]; for example, glass-fibre-reinforced polymer recovered from wind turbine blades in concrete [10] or combustion by-products in cement–soil composites [11]. However, recycled origin does not in itself guarantee an environmental benefit: concrete mix composition and the level of assessment can change the conclusions [12], which is also reflected in comparative LCAs of natural and recycled aggregate concrete [13]. Assessing numerous material alternatives with LCA requires detailed inventory data, specialist knowledge, and substantial time [14,15]. Machine learning has already been used to predict the mechanical performance of materials with waste-derived constituents [16] and, more broadly, material properties, for example, with graph neural networks [17] and multi-model, structure-agnostic frameworks [18].

In this context, ML may also support environmental assessment by estimating environmental indicators from existing LCA and EPD data. Reviews describe applications ranging from data estimation to direct prediction of impact indicators [14,19,20]. Related computational approaches address different parts of the assessment workflow. Hou et al. [21] developed a similarity-based method for estimating missing unit-process data, whereas Köck et al. [22] reviewed automation of life-cycle inventory compilation more broadly. These tasks should be distinguished from predicting life cycle impact assessment results directly from product-level data.

Predictive performance varies across impact indicators and product groups. Song et al. [23] used artificial neural networks (ANNs) to screen the life-cycle impacts of chemicals, reporting R^2^ values of 0.87 for Eco-indicator 99 and 0.48 for global warming. For construction products, Koyamparambath et al. [24] combined natural language processing with Random Forest (RF) regression and obtained an R^2^ of 0.81 for GWP prediction from EPD data. Tajuddeen et al. [25] predicted the GWP of thermal insulation materials using density, thermal conductivity, and embodied energy as predictors. Multiple linear regression, least absolute shrinkage and selection operator (LASSO) regression, and linear-kernel support vector regression (SVR) outperformed XGBoost under the validation procedures they examined.

Baehr et al. [15] extended EPD-based prediction to multiple impact indicators using a hybrid ANN–residual Gaussian process regression (ANN–rGPR) approach. Their analysis highlighted the importance of information availability and reporting completeness for prediction uncertainty. Neupane et al. [26] compared ANN, SVR, decision tree, RF, and extreme gradient boosting (XGB) models using ÖKOBAUDAT data. RF and XGB achieved R^2^ values of approximately 0.90 on the held-out test set and approximately 0.81–0.83 on an independent external EPD dataset. SVR achieved an R^2^ of approximately 0.55 on the external validation set. These results provide useful context, although model scores from different datasets and validation designs are not interchangeable benchmarks.

In a subsequent study, Neupane et al. [27] examined 22,397 construction-product EPDs from multiple programme operators in the ECO Portal. Cleaning and harmonization increased the XGB test R^2^ from 0.30 to 0.88; subsequent hyperparameter optimization increased it to 0.90. In that experiment, data preparation therefore produced a larger improvement than additional tuning. Their Shapley additive explanations (SHAP) analysis also identified the programme operator as an influential predictor, which the authors interpreted as a proxy for methodological differences between EPD schemes. The study included use of secondary material (SM) among its predictors and used GWP data from multiple declared life-cycle modules, including module D.

Related work has also addressed end-of-life circularity. Vergés et al. [28] developed a hybrid autoencoder–predictor model to forecast end-of-life pathways from material composition. Predicting a recovery pathway is a different task from predicting its associated GWP.

Building on this literature, the present study differs from previous EPD-based GWP prediction studies [24,25,26,27] in four respects. First, module D GWP–total is modelled as a separate target, predicted only from production-stage (A1–A3) information of the same EPD, and the sensitivity to the choice among multiple declared module D scenarios is examined in [27], where module D values were among several declared life-cycle modules rather than a separate prediction task. Second, the additional predictive information provided by declared secondary material use is quantified explicitly by ablation with bootstrap confidence intervals, rather than inferred from predictor importance alone. Third, the relationship between SM and module D GWP is analyzed within reference units and material groups and on a per-kilogram basis, which shows how pooling heterogeneous declarations can reverse the apparent direction of an association. Fourth, the robustness of the results is assessed with alternative outlier handling, repeated nested cross-validation, and validation grouped by manufacturer, product category, and programme operator, which indicates how far the models generalize beyond the records used for training.

The distinction between secondary material use and recyclability is essential to this analysis. SM is a resource-use indicator reported in kilograms per declared or functional unit; it does not, by itself, express the mass percentage of recycled constituents in a finished product [5]. Secondary material use should therefore be distinguished from both recycled-content percentage and end-of-life recyclability.

Module D reports potential environmental benefits and loads beyond the system boundary associated with reuse, recycling, and energy recovery [5,29]. Its values depend on the declared assumptions and should not be interpreted solely as a recycling credit. Anderson et al. [29] examined how EPDs report end-of-life and Module D data and scenarios, while Cheong et al. [30] assessed alternative end-of-life scenarios for curtain wall façades. These studies highlight the importance and variability of end-of-life assumptions.

This study evaluates ML regression models for GWP–total using ÖKOBAUDAT data, with particular attention to both the product stage and module D. It addresses four research questions:

(1) How accurately can A1–A3 GWP–total be predicted from EPD resource-use and waste indicators and declaration metadata, for all products and for products with declared secondary material use (SM > 0)?
(2) How much additional predictive information does SM provide beyond the other environmental indicators and EPD metadata?
(3) How are production-stage indicators, including SM, associated with module D GWP–total, and do these associations persist within reference units and material groups?
(4) To what extent can module D GWP–total be predicted from A1–A3 indicators and product metadata, and how well do these predictions generalize to unseen manufacturers and product categories?

Throughout the paper, “recycling potential” in the title refers to module D GWP–total, i.e., the declared potential benefits and loads beyond the system boundary [5], not to the end-of-life recyclability of a product.

## 2. Materials and Methods

**Figure 1:**
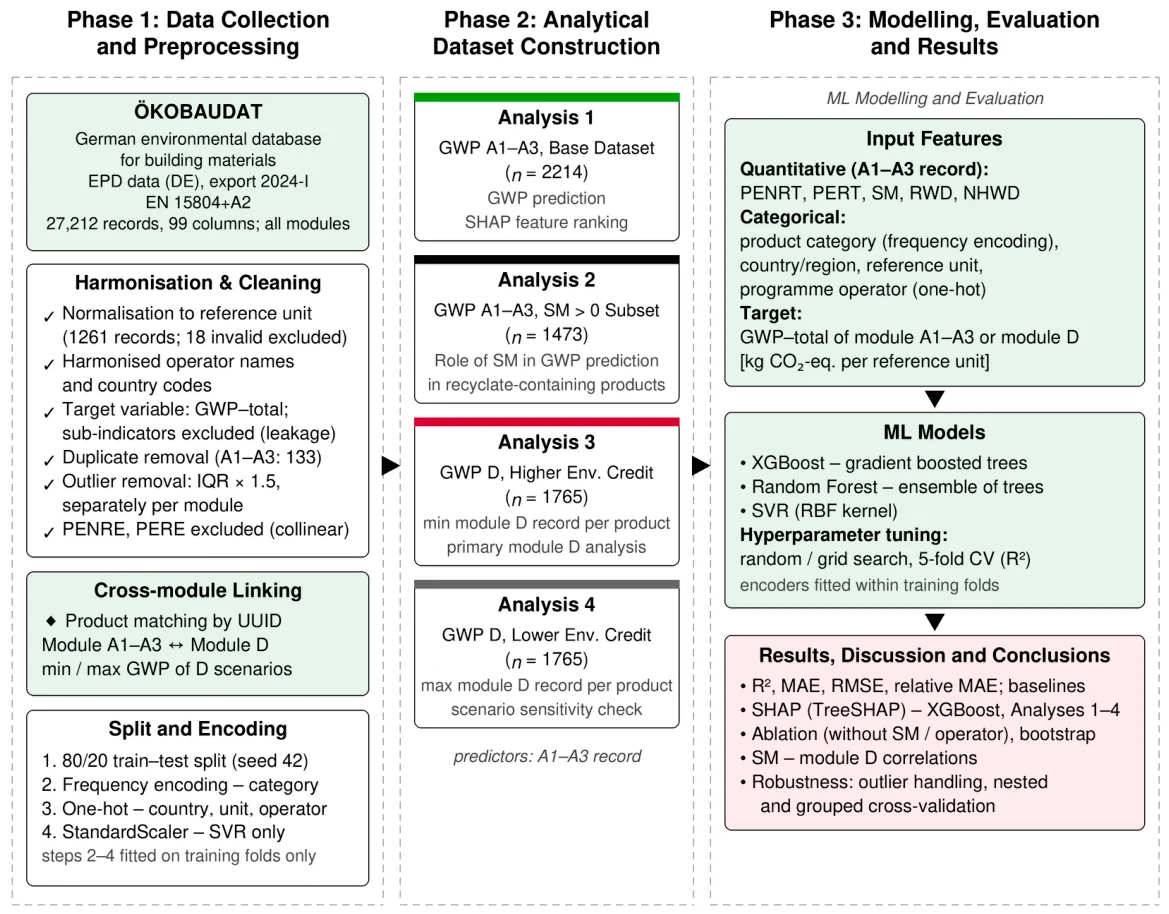
Study workflow. Own elaboration.

The study comprised three phases (Figure 1): (i) acquisition, harmonization, and cleaning of EPD data from ÖKOBAUDAT; (ii) construction of four analytical datasets corresponding to the prediction tasks; and (iii) training, optimisation, evaluation, and interpretation of machine-learning models. All stages were implemented within a single scripted computational pipeline. After each step, the pipeline recorded sample sizes, filtering thresholds, and selected parameters, allowing the results to be reproduced in full.

### 2.1. Data Source and Dataset Description

Data were obtained from ÖKOBAUDAT [7], a public database of EPDs for construction products maintained by the German Federal Ministry for Housing, Urban Development, and Building. The 2024-I database export, downloaded on 6 June 2026, contained 27,212 records and 99 columns. Each record represents one life-cycle module or one module scenario of a specific EPD dataset identified by a unique identifier (UUID). For the purposes of this study, the term “reference unit” (RU) is used as an umbrella term covering the declared unit or functional unit reported in EPDs, to which the indicator values refer (e.g., m^2^, kg, pcs, m^3^, m, or MJ). The records contain metadata including the product name and category, country or region, dataset type, EPD programme operator, reference unit, and reference quantity, as well as physical product properties, and impact and resource-flow indicators reported in accordance with EN 15804+A1 [31] or EN 15804+A2 [5].

The study addressed two prediction tasks. The first focused on the production stage (A1–A3 modules: A1—raw material supply; A2—transport; A3—manufacturing; cradle-to-gate), which accounts for the largest share of GWP–total in most construction-product categories and is characterised by the highest data completeness in EPD databases [6]. The second addressed module D, which covers potential benefits and loads beyond the system boundary arising from reuse, material recovery, recycling, and energy recovery. In this task, module D GWP–total predictions relied on information available for A1–A3 in the same EPD. This approach provided a basis for assessing how far production-stage characteristics capture a product’s environmental potential after the end of its life cycle, a question rarely examined using ML [28].

The target in both tasks was GWP–total, the total global warming potential defined in EN 15804+A2, expressed in kg CO_2_eq. per reference unit. This indicator was available for 26,270 database records. A further 114 records were excluded because their indicator values were reported exclusively in accordance with EN 15804+A1, the earlier version of the standard. Among the 2610 datasets (unique UUIDs) that reported A1–A3 GWP–total according to EN 15804+A2, 283 (10.8%) had no module D GWP–total value: 274 had no module D record and 9 had a module D record without a GWP–total value. In contrast, because PENRT, PERT, RWD, and NHWD are calculated in the same LCA model as GWP–total, an EPD that reports them normally also reports A1–A3 GWP–total. The realistic use cases are therefore the estimation of missing module D values from the A1–A3 indicators of the same EPD and plausibility checks of declared values, rather than filling gaps in A1–A3 GWP–total (Section 4).

Particular attention was given to SM, which, according to EN 15804+A2, represents the mass of secondary materials used as input to the product system, expressed in kg per reference unit [5]. An SM value greater than zero was treated as an indicator of declared secondary material use rather than as a complete description of product composition or recyclability. SM is also relevant to module D because potential benefits and loads beyond the system boundary are determined from the net flow of secondary materials [5]. The quantity entering the system is therefore considered when determining the quantity leaving the system for which potential benefits or loads may be reported in module D [5]. SM may thus matter for module D results, even though it does not itself describe a product’s end-of-life recycling potential. Section 3.4 presents the distribution of SM in the analyzed datasets.

### 2.2. Data Preparation

Data preparation comprised metadata harmonisation, normalisation to the reference unit, removal of duplicates and outliers, and construction of the analytical datasets. These procedures addressed the EPD data-quality dimensions identified by Neupane et al. [27], particularly completeness, methodological consistency, and geographical representativeness.

#### 2.2.1. Data Harmonisation and Normalisation

Harmonisation and normalisation involved the following steps:

- Reference-quantity validation. Records with non-positive or non-numeric reference quantities were excluded from the dataset (18 records), resulting in a final dataset of 27,194 records for further analysis.
- Normalisation to the reference unit. In 1261 records, the reference quantity differed from 1 (e.g., a declaration for 1000 kg or 10 m^2^). The models therefore included the reference unit as a categorical predictor.
- Harmonisation of EPD programme operator names and geographical codes.
- Exclusion of variables causing information leakage. The candidate predictor set excluded the components of GWP–total (GWP–fossil, GWP–biogenic, and GWP–luluc).

#### 2.2.2. Removal of Duplicates and Outliers

The 27,194 valid records yielded 2607 A1–A3 records with a reported GWP–total value. Only aggregated A1–A3 records were used; 369 records that declared modules A1, A2, and A3 only separately were not included. Records with identical values for the target and all five quantitative predictors were classified as duplicates, arising in part from repeated publication of the same dataset. Retaining only the first occurrence removed 133 records. An audit of the removed records (Table S3) showed that 7 had identical metadata (likely republication), 117 differed only in name while sharing the same product category, reference unit, and operator (product variants declared using a shared LCA profile), and 9 also differed in product category. These records therefore do not necessarily represent export errors. Only one occurrence of each profile was retained to prevent identical predictor and target vectors from appearing in both the training and test sets. No repeated UUIDs were found, leaving 2474 unique A1–A3 EPDs. The module D subset comprised 3073 records with a GWP–total value. Because an EPD may report several module D scenarios, module D records with repeated UUIDs remained in the dataset at this stage.

Target outliers were identified using the interquartile range (IQR) [32]. Specifically, the procedure used Tukey’s criterion based on the IQR. This method was selected because it is relatively insensitive to individual extreme values and does not assume a normal distribution. Unlike methods based on the mean and standard deviation, it defines outlier thresholds from quartiles. Individual extreme observations therefore have less influence on the identification procedure itself. The method is also simple, transparent, and requires no additional parameter tuning. The procedure treated each module separately used IQR to flag observations for manual review. Observations outside [Q1 − 1.5·IQR; Q3 + 1.5·IQR] were classified as outliers, where Q1 and Q3 are the first and third quartiles of the GWP–total distribution. For A1–A3 (Q1 = 1.54; Q3 = 90.49; IQR = 88.95 kg CO_2_eq.), the interval was [−131.88; 223.91] kg CO_2_eq. Filtering removed 260 records (10.5%), leaving 2214 EPDs. For module D (Q1 = −9.81; Q3 = −0.24; IQR = 9.57 kg CO_2_eq.), the interval was [−24.17; 14.12] kg CO_2_eq. Filtering removed 500 records (16.3%); the remaining 2573 records represented 2232 EPDs. Filtering preceded the train–test split and used the full dataset for each module. Exceeding an IQR threshold does not indicate a declaration error, and exclusions were unevenly distributed across reference units and material groups (e.g., 27.0% of A1–A3 records declared per m^3^ and 57.5% of A1–A3 records for wood products; Table S4). Section 3.5 and Table S15 report the sensitivity of the results to this choice, and Section 4 discusses its consequences.

#### 2.2.3. Construction of the Analytical Datasets

The cleaned data formed the basis for four analytical datasets (Table 1), referred to hereafter as Analyses 1–4:

- Analysis 1—GWP A1–A3, Base Dataset–all 2214 A1–A3 EPDs, with A1–A3 GWP–total as the target;
- Analysis 2—GWP A1–A3, SM > 0 Subset–a subset of 1473 EPDs with SM > 0, used to assess prediction performance and the importance of SM in products that use secondary materials;
- Analysis 3—GWP D, Higher Env. Credit and Analysis 4—GWP D, Lower Env. Credit–1765 EPDs for which A1–A3 records were linked to module D records by UUID (inner join). Predictors came from the A1–A3 record, and module D GWP–total was the target.

More than one module D scenario was reported for 175 of the 1765 linked EPDs, and GWP–total values differed between scenarios in 170 cases. Two dataset variants were created to avoid arbitrary scenario selection. For EPDs with multiple scenarios, the lowest GWP–total value was assigned to Analysis 3—GWP D, Higher Environmental Credit, whereas the highest value was assigned to Analysis 4—GWP D, Lower Environmental Credit. Both variants contained the same EPDs and predictors; only the target values for EPDs with multiple scenarios differed. Analysis 3 is treated as the primary module D analysis and Analysis 4 as a scenario sensitivity check because the two targets were identical for 1595 of the 1765 EPDs (90.4%). In both variants, most module D GWP–total values were negative (Table 1), indicating potential environmental benefits beyond the system boundary.

Not all records are EPDs in the narrow sense. Besides manufacturer-specific EPDs, ÖKOBAUDAT contains average, generic, representative, and template datasets, which are not product-specific declarations. In Analysis 1, 1045 records (47.2%) were specific, 727 (32.8%) average, 360 (16.3%) generic, 49 (2.2%) template and 33 (1.5%) representative datasets (Table S1). All were reported in the same EN 15804+A2 format and were retained; for brevity, the term “EPD” refers to all of these records. Performance by dataset type is reported in Section 3.5 and Table S20.

### 2.3. Predictor Selection and Encoding

**Figure 2:**
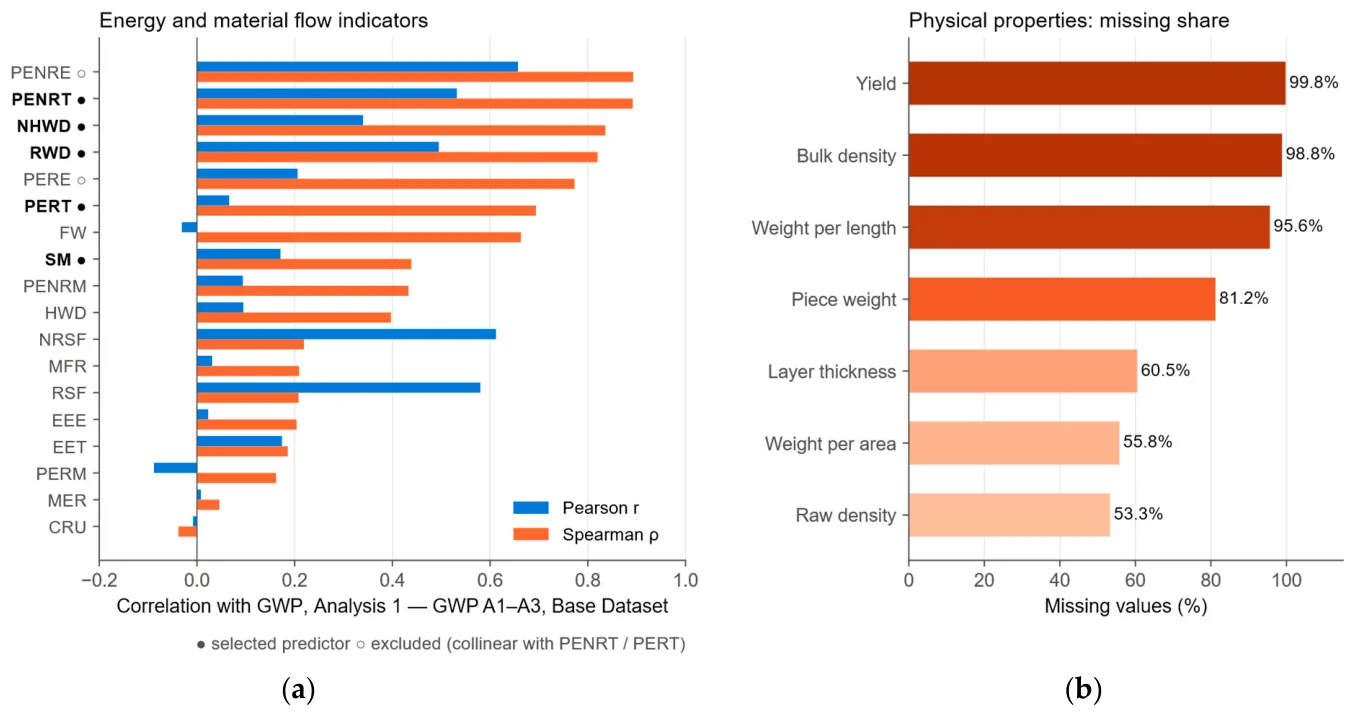
Preliminary assessment of candidate predictors in Analysis 1—GWP A1–A3, Base Dataset (*n* = 2214): (**a**) Pearson (r) and Spearman (ρ) correlations between energy- and material-flow indicators and GWP–total. Predictors selected for the models are shown in bold; ○ denotes indicators excluded because of collinearity. (**b**) Proportion of missing values for physical product properties. Own elaboration.

Candidate predictors included energy and material flow indicators reported in EPDs, physical product properties, and declaration metadata. The analysis adopted the quantitative predictor set used in [27], where PENRT, PERT, SM, NHWD, and RWD provided the most information for GWP–total prediction from EPD data. A suitability check examined data completeness and the Pearson (r) and Spearman (ρ) correlations of candidate variables with the target in Analysis 1 (Figure 2). This check used the entire dataset because the predictors had been specified a priori, rather than selected from its results.

Except for SM, the selected indicators were among those with the strongest monotonic associations with GWP–total (Figure 2a): PENRT (ρ = 0.892), NHWD (ρ = 0.836), RWD (ρ = 0.820) and PERT (ρ = 0.694). Their Pearson coefficients were considerably lower (r = 0.066–0.532), indicating non-linear relationships and a strong influence of high-value observations. SM (r = 0.171; ρ = 0.439) remained in the predictor set regardless of its correlation strength because its role is central to the study. PENRE, PENRM, PERE, and PERM, whose sums form PENRT and PERT, were excluded to avoid duplicating information. Collinearity among the energy components was very high (r = 0.960 for the PENRE–PENRT pair and r = 0.910 for the PERE–PERT pair). HWD, MFR, MER, EEE, EET, and CRU showed weak monotonic associations with the target (ρ ≤ 0.40). The relatively high Pearson coefficients for RSF and NRSF (r ≈ 0.6), despite low ρ values (≈0.2), reflected a few high-value observations: approximately 80% of EPDs reported zero for these indicators. These indicators were therefore excluded. FW (ρ = 0.663) was omitted to maintain comparability with [27].

Physical product properties (including bulk density, mass per unit area, layer thickness, and unit mass) were excluded because missingness ranged from 53.3% to 99.8% (Figure 2b), consistent with the observations for ÖKOBAUDAT in [26]. Given the heterogeneity of the products, mean or median imputation was considered unjustified.

Four categorical predictors described the declaration context: product category, country or region, reference unit, and EPD programme operator (Table 2). Product category, recorded using the three-level ÖKOBAUDAT classification (221 unique values in the Analysis 1—GWP A1–A3, Base Dataset), was frequency-encoded: each category was replaced with its relative frequency in the training set. Categories absent from training received a value of 0. This encoding does not preserve category identity. Equally frequent categories receive the same value, and the 206 categories in the Analysis 1—GWP A1–A3, Base Dataset training set mapped to 31 distinct values (Table S10). Section 3.5 and Table S20 report a sensitivity analysis with one-hot encoding of product category and of the first-level material group. The importance of “product category” therefore represents the importance of category frequency in the dataset. Variables with moderate numbers of levels, country, or region (34 levels), reference unit (6), and EPD programme operator (7) were one-hot encoded, ignoring levels absent from training. The resulting predictor matrix for Analysis 1—GWP A1–A3, Base Dataset had 53 columns. The few missing quantitative predictor values (≤ 1% records: RWD–20, NHWD–3, SM–1) were replaced with zero, although a missing report is not equivalent to a zero flow. Given the small number of missing values, the effect of this choice was considered limited. Correlations computed using only complete observation pairs in the module D datasets agreed with the main results (Table S13). No categorical values were missing.

The pipeline incorporated all transformations (frequency encoding, one-hot encoding, and standardisation) as components (scikit-learn Pipeline) and fitted them exclusively to training data within each cross-validation fold and to the full training set before test evaluation. This prevented information from the validation and test sets from entering the learning process.

### 2.4. Machine-Learning Models

Based on a review of ML applications for predicting environmental impacts from EPD data [14,19,20], we selected three regression algorithms representing different learning approaches: XGBoost, RF, and SVR.

XGBoost [34] builds an ensemble of decision trees sequentially. Each successive tree corrects the residual errors of the preceding trees, while L1 and L2 regularisation limit model complexity. In studies using EPD data from ÖKOBAUDAT and other databases, XGBoost achieved the highest accuracy among the algorithms tested (R^2^ = 0.88–0.90 on the test sets) [26]. A further advantage is the availability of the exact TreeSHAP algorithm for model interpretation [35].

Random Forest [36] combines decision trees trained on bootstrap samples, with a random subset of predictors considered at each node. Its prediction is the mean of the individual tree predictions. Averaging reduces overfitting and stabilises predictions. In EPD-based studies, RF achieved R^2^ = 0.81 for GWP–total [24] and was usually outperformed only by XGBoost [26]. Including RF made it possible to assess whether the findings were consistent across two different tree-based ensemble methods.

SVR [37] uses a kernel function to map predictors into a multidimensional feature space and fits a regression function that does not penalise errors smaller than ε. It differs from tree-based methods and is sensitive to variable scale. In [27], which used ECO Portal data, SVR generalised less well on heterogeneous EPD data. In a narrower, more homogeneous product group (insulation materials), however, it achieved competitive performance (R^2^ = 0.82), with the best results obtained using a linear kernel [25]. SVR served as a contrasting model. A radial basis function (RBF) kernel was selected to capture potential non-linear relationships in the heterogeneous dataset. Table S7 compares its performance with linear models. Both predictors and the target were standardised within the pipeline, and predictions were transformed back to their original units before calculating error metrics. Tree-based models used unscaled predictors because decision-tree splits are invariant to monotonic variable transformations.

Artificial neural networks were not included because previous studies reported lower performance than tree-based ensemble methods on EPD datasets of similar size [26], and their full potential generally requires substantially larger training datasets [15]. Linear models, which are better suited to narrow, homogeneous product groups [25], were included as baselines (Section 2.5).

#### Hyperparameter Optimisation

Each model was tuned independently for each of the four datasets using only the training data and five-fold cross-validation with shuffled K-Fold splits, with the mean coefficient of determination (R^2^) used as the selection criterion. For XGBoost and Random Forest, which have relatively large hyperparameter spaces, random search (RandomizedSearchCV) [38] over 50 and 40 combinations, respectively. For SVR, exhaustive grid search (GridSearchCV) evaluated 100 combinations of C, ε, and γ. After selecting the best configuration, we refitted the model on the full training set. Table 3 lists the search spaces and selected hyperparameters.

To quantify the benefit of tuning, we also calculated cross-validation performance for each model’s initial configuration. In [27], hyperparameter tuning improved R^2^ by only 0.02, whereas data-quality improvements increased R^2^ by 0.58. In line with those findings, the study prioritised data preparation and treated hyperparameter optimisation as a supplementary step.

### 2.5. Model Validation and Evaluation

Each analytical dataset was randomly divided into training (80%) and test (20%) sets using a fixed random seed (Table 1). The test set was used only for final evaluation, not for variable encoding, model fitting, or hyperparameter selection. The split was not stratified by product category because 56 of the 221 categories contained a single record and 128 contained fewer than five records.

Model performance was assessed using three metrics: the coefficient of determination (R^2^), mean absolute error (MAE), and root mean squared error (RMSE), defined in Equations (1)–(3):

R^2^ = 1 − Σ_i_ (y_i_ − ŷ_i_)^2^/Σ_i_ (y_i_ − ȳ)^2^ (1)

MAE = (1/n) Σ_i_ |y_i_ − ŷ_i_| (2)

RMSE = √[(1/n) Σ_i_ (y_i_ − ŷ_i_)^2^] (3)

where y_i_ is the observed value, ŷ_i_ the predicted value, ȳ the mean observed value, and n the number of observations. R^2^ describes the proportion of target variance explained by the model: 1 indicates a perfect fit, 0 indicates performance equivalent to predicting the mean, and negative values indicate worse performance. MAE and RMSE are expressed in kg CO_2_eq. per reference unit; RMSE penalises large errors more strongly than MAE.

For each model, the reported metrics comprised training R^2^, the mean and standard deviation of five-fold cross-validation R^2^ for the selected hyperparameter configuration, and test R^2^, MAE, and RMSE. Training R^2^ values were compared with cross-validation and test-set results to assess potential overfitting. Because the cross-validation score for the selected configuration comes from the same process used to choose hyperparameters, it may be slightly optimistic. The test set provides an independent evaluation. Models were evaluated separately for all four datasets to compare performance across all products and products with SM > 0, and between A1–A3 and module D.

To assess the added value of ML, the evaluation included four simple baselines using the same split: the median target within each reference unit; the median within each category × unit combination (for groups with at least three training records, otherwise the unit median); linear regression using PENRT and reference unit; and linear regression using all predictors (Table S7). Uncertainty in performance differences between XGBoost and Random Forest, as well as between the ablation variants, was estimated using paired bootstrap resampling with 2000 repetitions on the common test set; 95% percentile intervals were reported (Table S8). A difference was described as not statistically conclusive when its 95% bootstrap interval included zero. Scale-free errors were reported as relative MAE (MAE divided by the mean absolute observed value) and as the ratio of model MAE to that of the category × unit median baseline, overall and by reference unit (Table S5). For A1–A3, a log–log linear baseline, ln(GWP–total) ln(PENRT) + reference unit, was also fitted to records with positive GWP–total and PENRT (Table S7). Additional summaries reported performance by reference unit (Table S5), material group (Table S14), presence of the product category in training (Table S6), and the structure of module D variants within EPD groups (Table S9). A single split with a fixed seed ensures reproducibility but does not quantify variation due to the choice of split.

The robustness of the main results was examined in five additional analyses, performed for Analyses 1–3 (Analysis 4 differed from Analysis 3 only in the targets of 170 EPDs):

- Outlier handling (Table S15): the models were refitted without outlier removal, with the IQR filter applied to the training set only (globally, and separately for each reference unit, because most category × unit groups were too small for stable quartiles), and without outlier removal but with a sign-preserving inverse hyperbolic sine (asinh) transformation of the target. For module D, the scenario minimum was selected before filtering. All variants were split before outlier handling and evaluated on the same test records.
- Repeated nested cross-validation (Table S19): 5-fold outer cross-validation repeated three times, with a random search over the hyperparameter spaces of Table 3 (20 candidates, 3-fold inner cross-validation) inside each outer training fold, for XGBoost and Random Forest. Nested cross-validation was applied to the datasets after the global IQR filtering (Section 2.2.2), so the filter itself was not refitted within the outer folds.
- Grouped validation: 5-fold GroupKFold with groups defined by declaration owner (manufacturer) or product category (Table S17), and leave-one-operator-out validation (Table S16).
- Category encoding and dataset type (Table S20): one-hot encoding of product category, or of the first-level material group, instead of frequency encoding and performance by dataset type.
- Systematic bias (Table S18, Figure S1): signed errors by reference unit and material group. Except for nested cross-validation, these analyses used the tuned hyperparameters of the main models (Table 3), which had been selected on the random training split. The grouped and leave-one-operator-out validations therefore assess how a fixed model configuration transfers to unseen groups, not the complete model selection procedure.

### 2.6. Predictor Importance and the Role of SM

Relative predictor importance in XGBoost was assessed using SHAP values (SHapley Additive exPlanations) [39]. A SHAP value φ_ij_ quantifies the contribution of predictor j to the prediction for observation i relative to the model’s expected value φ_0_. The prediction is the sum of these contributions (Equation (4)):

ŷ_i_ = φ_0_ + Σ_j_ φ_ij_ (4)

SHAP values were computed using the exact TreeSHAP algorithm [35] implemented in XGBoost through the pred_contribs prediction option. This implementation was used because the TreeExplainer module of the SHAP library (version 0.49.1 used in this study) did not support the XGBoost 3.x model format. Calculation accuracy was verified by checking additivity: the maximum difference between the sum of SHAP contributions and the model prediction did not exceed 0.0004 kg CO_2_eq.

Global predictor importance was calculated as the mean absolute SHAP value on the test set. For one-hot-encoded variables, SHAP values were summed across all columns corresponding to a given variable. The resulting importance values were then normalised so that their sum equalled 1 within each dataset (Equation (5)):

I_g_ = Σ_j∈g_ (1/n) Σ_i_ |φ_ij_|, Ĩ_g_ = I_g_/Σ_h_ I_h_ (5)

where g denotes an original variable (i.e., EPD operator), and j_∈_g denotes the predictor-matrix columns representing that variable.

The normalised value Ĩ_g_ allows comparison of predictor-importance patterns across datasets with different target scales. The direction of the effects of quantitative predictors was assessed by examining the distribution of SHAP values in relation to predictor values. For categorical predictors, the analysis examined SHAP values summed across the columns representing each variable. SHAP describes how a model uses information; it does not support causal inference. The measure I_g_ does not allow opposite sign contributions within a variable to cancel. An additional check used the alternative measure (Equation (6)).

J_g_ = (1/n) Σ_i_ |Σ_j∈g_ φ_ij_| (6)

The rankings obtained with the two measures agreed (Spearman’s rank correlation 0.93–1.00); Table S11 reports the unnormalised values. Normalisation permits comparisons of predictor shares, not absolute variable importance across datasets. The comparison also included normalised XGBoost gain, summed across one-hot-encoded columns (Table S12).

A low SHAP value does not establish that a variable provides no additional predictive information because correlated predictors may capture part of that information. An XGBoost ablation analysis therefore examined this topic with hyperparameters held fixed. For each dataset, the study compared the base model, a model without SM, and a model without the EPD programme operator and all its one-hot columns. The ablation variants retained the base model’s selected hyperparameters, train–test split and cross-validation folds. Differences in ΔR^2^ therefore primarily reflected removal of the variable. Changing the number of columns could affect only random column sampling in XGBoost, so small differences require caution. The ablation also covered the programme operator because the literature identifies it as a possible proxy for methodological differences between EPD programmes [27]. Differences were expressed as ΔR^2^ and relative changes in mean squared error (MSE), with uncertainty estimated by bootstrap (Table S8). ΔR^2^ was defined as the R^2^ of the reduced model minus that of the base model. Leave-one-operator-out validation (Section 2.5) complemented the operator ablation. Fixed-configuration ablation does not determine whether a retuned model without a variable would perform worse.

SM was also characterised using descriptive statistics (counts of EPDs with SM = 0 and SM > 0, medians, quartiles, means, and maximum values). The correlation analysis assessed relationships between quantitative A1–A3 predictors and module D GWP using Pearson (linear) and Spearman (monotonic) correlations in the Analysis 3—GWP D, Higher Env. Credit and Analysis 4—GWP D, Lower Env. Credit datasets (*n* = 1765). Statistical significance was assessed at α = 0.05. One missing SM value was replaced with zero, consistent with the modelling procedure.

SM and GWP–total are reported per reference unit, so the overall correlation may therefore reflect differences in scale associated with the reference unit and product size rather than an association between SM (A1-A3) and GWP–total (D). The relationship between these variables was analysed separately within material groups and reference units. Spearman correlations were calculated for subsets containing at least 10 EPDs. As a sensitivity check, unit conversion to values per kg of product was performed using physical property data from the database. Conversion was possible for 1741 EPDs.

### 2.7. Implementation and Computational Reproducibility

The calculations were performed in Python 3.11 using pandas 2.3.3 and NumPy 2.2.6 (data processing), scikit-learn 1.7.2 [40] (RF, SVR, hyperparameter optimization, and cross-validation), XGBoost 3.2.0 [34] (XGBoost model and SHAP values), SciPy 1.15.3 (correlation analysis), and Matplotlib 3.11.2 (visualizations). A fixed random seed of 42 controlled all stochastic elements (data splitting, cross-validation subsets, random search, and ensemble algorithms).

Additional methodological details and supplementary analyses supporting Section 2, Section 3 and Section 4 are provided in the Supplementary Materials (Tables S1–S21 and Figure S1).

## 3. Results

### 3.1. GWP Prediction Performance for A1–A3

The study characterised the product population before model evaluation presented in (Table S2). The largest material groups were mineral products (706), building services (399), window and curtain–wall components (396), and plastics (228). The proportion of EPDs with SM > 0 ranged from 21.2% for coverings to 81.7% for metals. Reference units varied both between and within groups; for example, mineral products were declared per m^3^ (272), kg (270), m^2^ (159), and m (5). Module D linkage was available for 62–97% of EPDs in each group. EPDs for which the two module D target values differed were concentrated mainly in plastics (86) and mineral products (43).

The assessment of production-stage GWP prediction used Analysis 1—GWP A1–A3, Base Dataset and Analysis 2—GWP A1–A3, SM > 0 Subset datasets, the latter containing only records with positive secondary material use (SM > 0). Table 4 compares model performance.

For Analysis 1, XGBoost achieved the best predictive performance, with test R^2^ = 0.963, MAE = 5.30 and RMSE = 11.98; the MAE corresponded to 12.6% of the mean absolute observed value and to 0.33 of the MAE of the category × unit median baseline (Table S5). Random Forest performed similarly (R^2^ = 0.958), whereas SVR had a lower R^2^ (0.941). The R^2^ difference between XGBoost and Random Forest (0.005) was smaller than the bootstrap uncertainty (Table S8). Both models clearly outperformed the simple baselines: the category × unit median (R^2^ = 0.716) and linear regression with all predictors (R^2^ = 0.834) (Table S7).

Five-fold cross-validation confirmed the high and relatively stable performance of the tree-based models (XGBoost and Random Forest). Mean R^2^ was 0.937 ± 0.020 for XGBoost and 0.935 ± 0.022 for Random Forest. SVR had a lower mean R^2^ of 0.907 ± 0.037.

**Figure 3:**
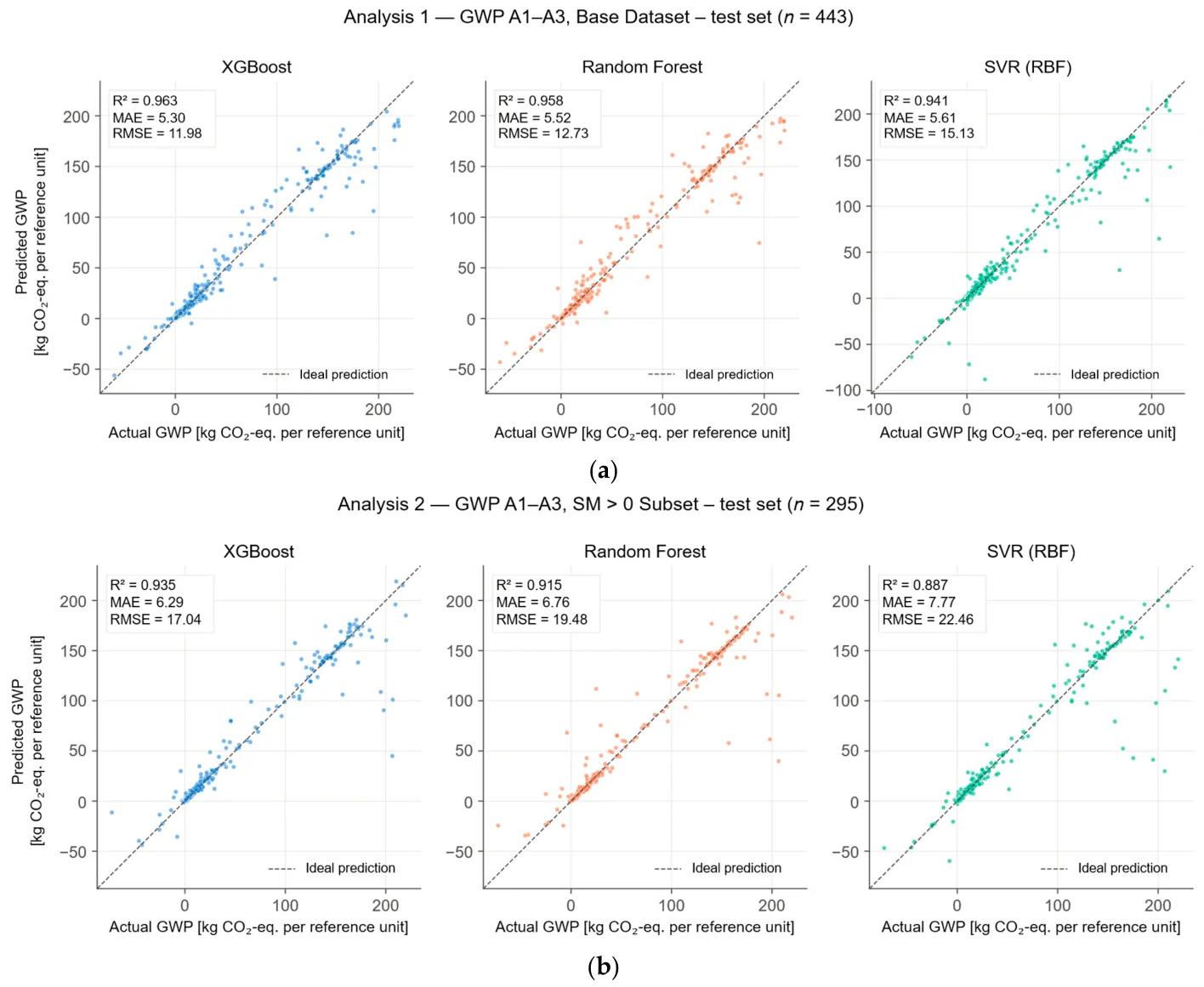
Observed and predicted test-set GWP–total values for (**a**) Analysis 1—GWP A1–A3, Base Dataset (*n* = 443) and (**b**) Analysis 2—GWP A1–A3, SM > 0 Subset (*n* = 295). Own elaboration.

Tree-based models again performed best on Analysis 2. Test R^2^ was 0.935 for XGBoost and 0.915 for Random Forest, compared with a lower value for SVR (R^2^ = 0.887). This was the only dataset in which the XGBoost–Random Forest difference was supported by the bootstrap interval (ΔR^2^ = 0.020; 95% CI 0.002 to 0.041; Table S8); the relative MAE of XGBoost was 11.9%. Mean five-fold cross-validation R^2^ was 0.964 ± 0.007 for XGBoost and 0.954 ± 0.011 for Random Forest. Figure 3 shows agreement between observed and predicted GWP for Analysis 1 (Figure 3a) and Analysis 2 (Figure 3b).

Training R^2^ values of the tree-based models (0.985–0.999) exceeded cross-validation scores. Performance varied across reference units: for XGBoost on Analysis 1, R^2^ ranged from 0.785 for products declared per kg to 0.988 for those declared per m, and relative MAE from 8.0% (m^3^) to 36.8% (kg) (Table S5). Across material groups, R^2^ ranged from 0.637 for metals to 0.969 for mineral products (Table S14). For the 17 test observations whose product categories were absent from training, R^2^ was 0.743, compared with 0.967 for categories represented in training (Table S6). The log–log linear baseline reached R^2^ = 0.627 on the original scale and 0.925 on the log scale, compared with 0.962 and 0.794 for XGBoost on the same 425 test records with positive GWP–total and PENRT (Table S7).

### 3.2. Prediction of Module D GWP

After evaluating A1–A3 GWP prediction, we assessed the prediction of module D GWP using two linked-data variants: Analysis 3 and Analysis 4. Both contained the same 1765 EPDs but differed in the selected module D record. Analysis 3 used the lowest GWP_D among records retained after IQR filtering and is the primary module D analysis; Analysis 4 used the highest and is reported as a scenario sensitivity check. Table 5 presents model performance.

For Analysis 3, XGBoost (R^2^ = 0.791) and Random Forest (R^2^ = 0.775) achieved the highest test R^2^ values. SVR performed less well (R^2^ = 0.680). The XGBoost–Random Forest difference was within bootstrap uncertainty (ΔR^2^ = 0.015; 95% CI −0.015 to 0.044; Table S8). XGBoost achieved MAE = 1.14 (26.8% of the mean absolute observed value; 0.53 of the baseline MAE) and RMSE = 2.27, compared with MAE = 1.22 and RMSE = 2.35 for Ran-dom Forest. Mean five-fold cross-validation R^2^ was 0.725 ± 0.072 for XGBoost and 0.706 ± 0.062 for Random Forest.

For Analysis 4, XGBoost achieved the best test performance (R^2^ = 0.814), followed closely by Random Forest (R^2^ = 0.790). SVR had a lower score (R^2^ = 0.665). XGBoost achieved MAE = 1.18 and RMSE = 2.13, compared with MAE = 1.18 and RMSE = 2.26 for Random Forest. Mean five-fold cross-validation R^2^ was 0.719 ± 0.084 for XGBoost and 0.692 ± 0.073 for Random Forest.

**Figure 4:**
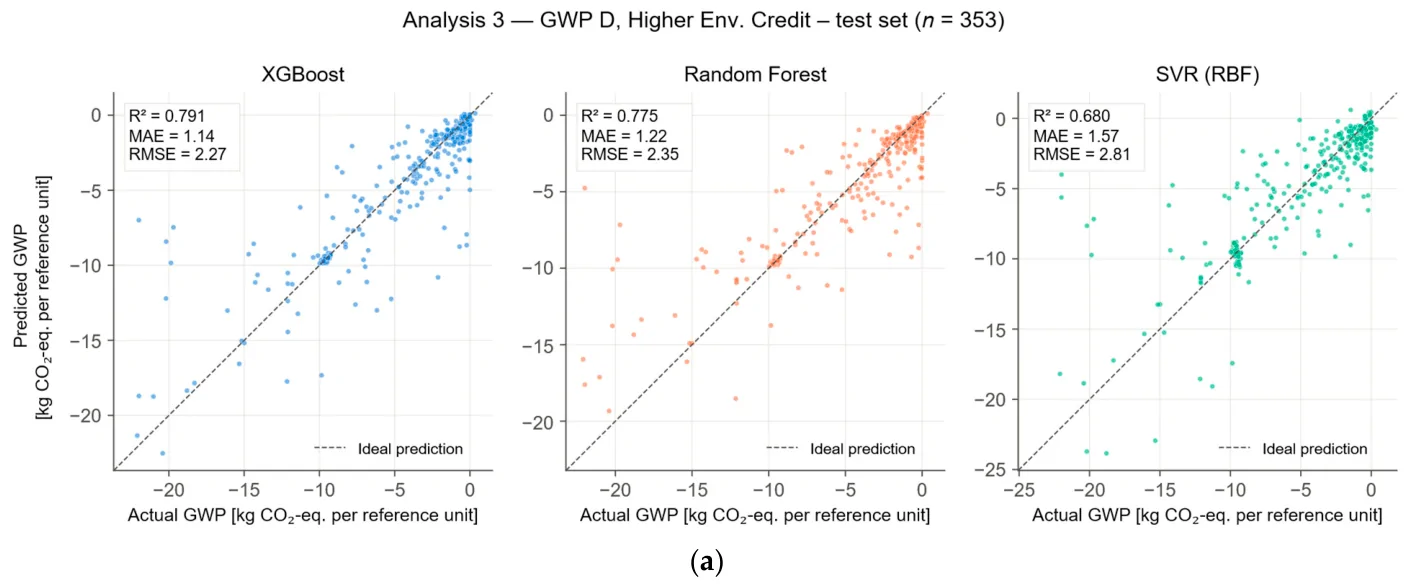

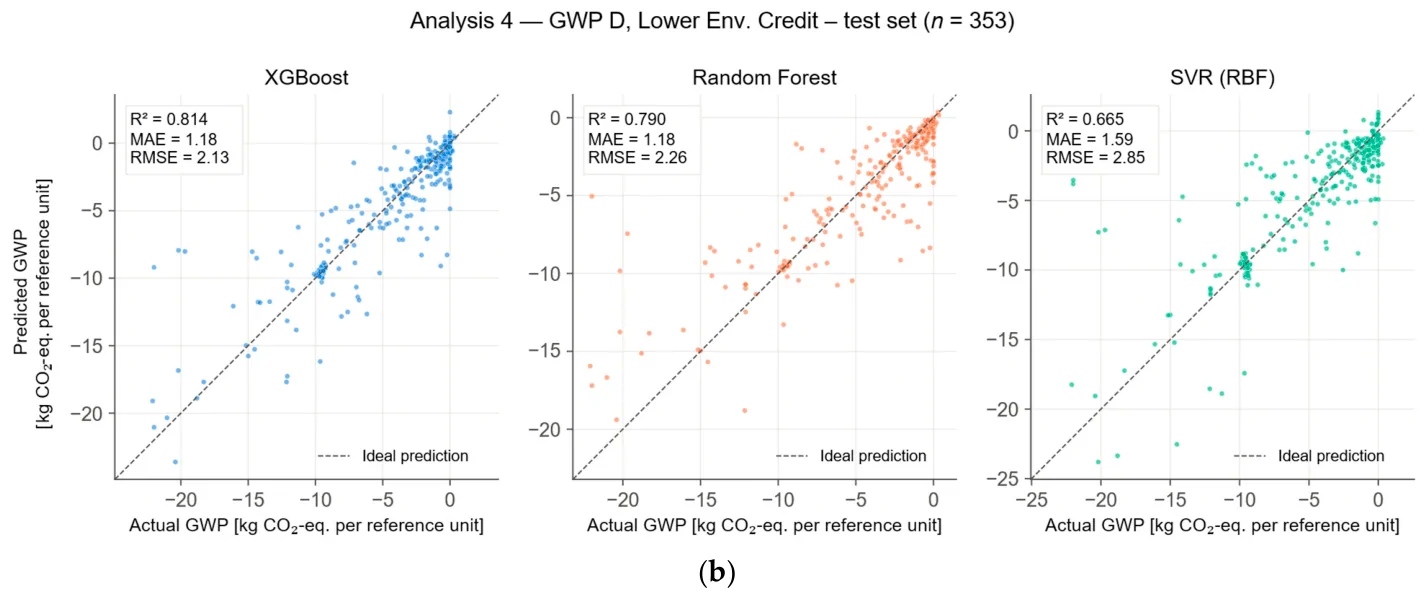
Observed and predicted module D GWP values on the test set (*n* = 353) for (**a**) Analysis 3—GWP D, Higher Env. Credit and (**b**) Analysis 4—GWP D, Lower Env. Credit. Own elaboration.

Agreement between observed and predicted module D GWP for individual models is shown in Figure 4 for Analysis 3—GWP D, Higher Env. Credit (Figure 4a) and Analysis 4—GWP D, Lower Env. Credit (Figure 4b).

Test R^2^ values exceeded cross-validation means (for XGBoost by 0.07–0.09), and cross-validation standard deviations were several times greater than for A1–A3. Training R^2^ (0.960–0.995) was considerably higher than cross-validation R^2^. ML models clearly outperformed the baselines, whose R^2^ did not exceed 0.475 (Table S7). XGBoost performed worst for products declared per kg (R^2^ = 0.296 for Analysis 3; Table S5). The two target variants were identical for 1595 of the 1765 EPDs (90.4%). Among the 170 EPDs for which they differed (25 test observations), R^2^ was 0.658 for Analysis 3 and 0.652 for Analysis 4. The predicted difference between the two variants averaged 1.23 kg CO_2_eq., compared with an observed difference of 1.95 kg CO_2_eq. Because the models were trained independently, the predicted GWP–total for Analysis 3 exceeded that for Analysis 4 in 163 of the 353 test cases (Table S9).

### 3.3. Predictor Importance in the Models

**Figure 5:**
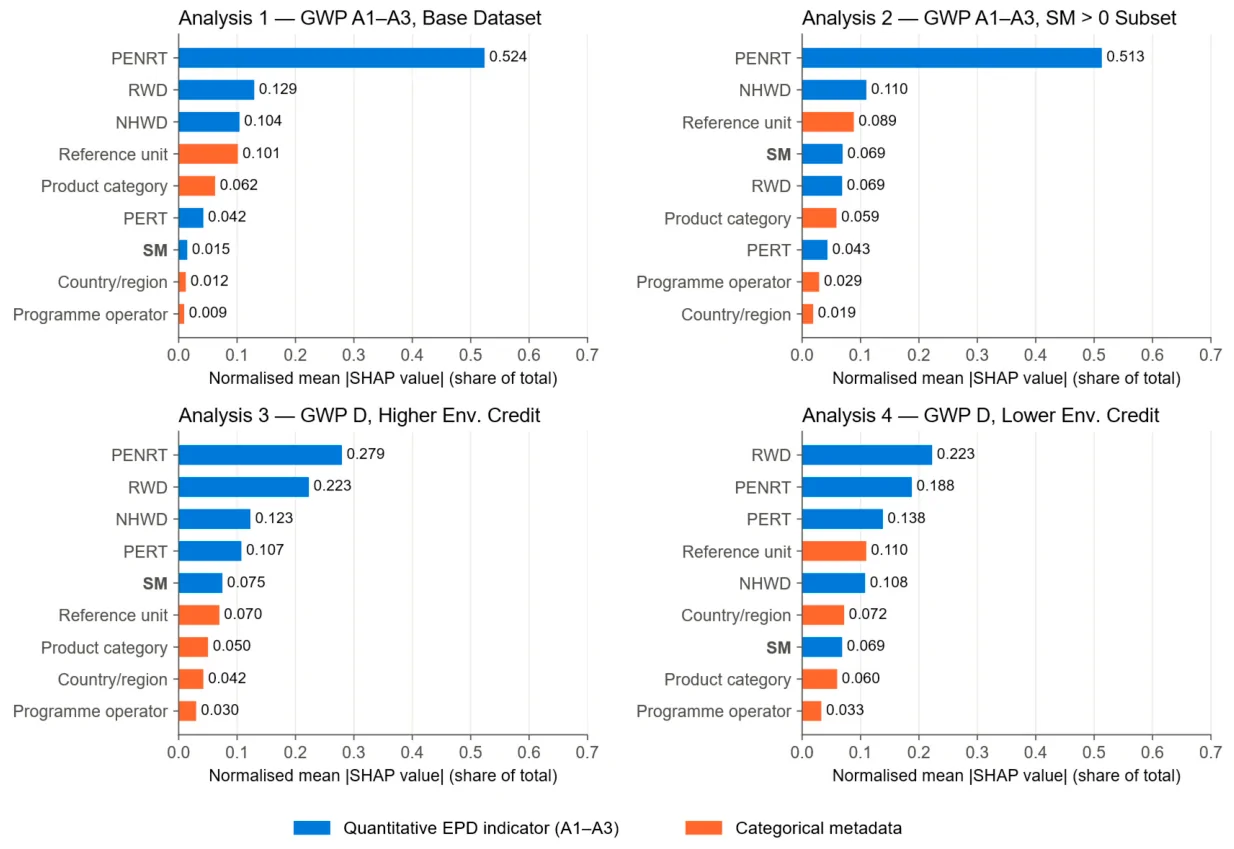
Normalised predictor importance based on XGBoost mean |φ_j| SHAP for Analysis 1—GWP A1–A3, Base Dataset, Analysis 2—GWP A1–A3, SM > 0 Subset, Analysis 3—GWP D, Higher Env. Credit and Analysis 4—GWP D, Lower Env. Credit. Own elaboration.

**Figure 6:**
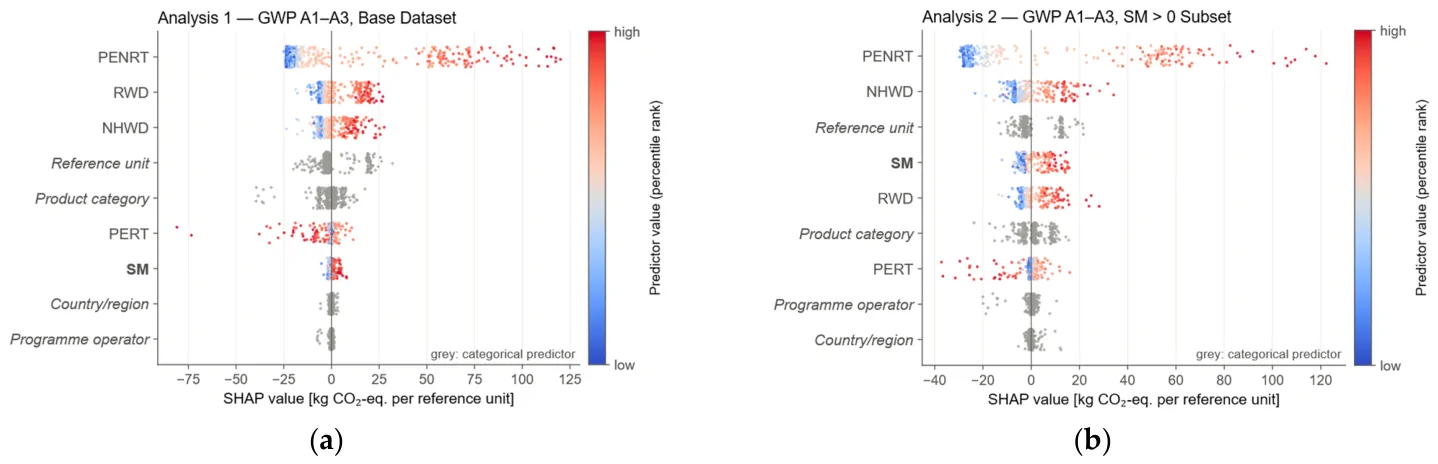

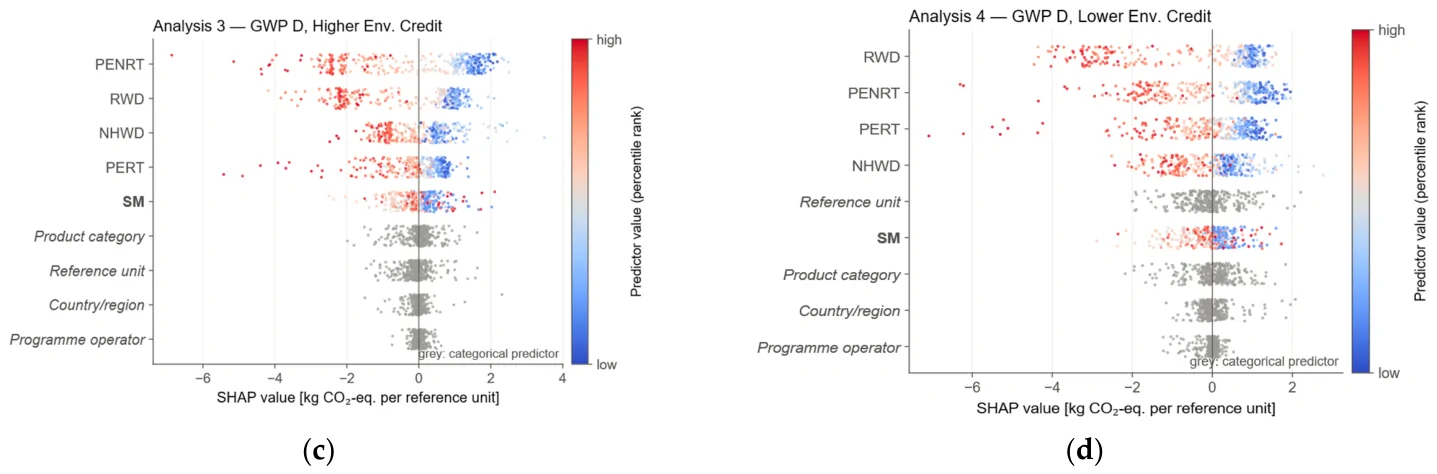
Test-set distributions of predictor SHAP values in the XGBoost models: (**a**) Analysis 1—GWP A1–A3, Base Dataset, (**b**) Analysis 2—GWP A1–A3, SM > 0 Subset, (**c**) Analysis 3—GWP D, Higher Env. Credit, and (**d**) Analysis 4—GWP D, Lower Env. Credit. Own elaboration.

The analysis then examined relative predictor importance using the normalised SHAP importance measure (mean |φ_j|) for XGBoost. Figure 5 compares predictor importance across Analysis 1—GWP A1–A3, Base Dataset, Analysis 2—GWP A1–A3, SM > 0 Subset, Analysis 3—GWP D, Higher Env. Credit and Analysis 4—GWP D, Lower Env. Credit. Figure 6 shows the distributions of quantitative and categorical predictor SHAP values across individual observations. Hereafter, “marginal” and “moderate” are descriptive terms. The predictor rankings remained consistent across methods of aggregating SHAP values for one-hot-encoded variables (Table S11). The importance of “product category” refers to its frequency-encoded representation (Section 2.3).

The two A1–A3 datasets showed similar relative predictor-importance patterns. In Analysis 1—GWP A1–A3, Base Dataset, PENRT had the highest mean |φ_j| SHAP (0.524), followed by RWD (0.129), NHWD (0.104), and reference unit (0.101). The corresponding values in Analysis 2—GWP A1–A3, SM > 0 Subset were 0.513, 0.069, 0.110, and 0.089. Other predictors had lower relative importance; the EPD programme operator (0.009 and 0.029) and country or region (0.012 and 0.019) had marginal importance. SM importance was 0.015 in Analysis 1—GWP A1–A3, Base Dataset and 0.069 in Analysis 2—GWP A1–A3, SM > 0 Subset. Higher PENRT, RWD, NHWD, and SM values were associated with higher predicted GWP. Categorical predictor SHAP values clustered near zero, except for reference unit and product category (Figure 6a,b).

Module D GWP models showed a different predictor-importance pattern. For Analysis 3—GWP D, Higher Env. Credit, PENRT had the highest mean |φ_j| SHAP (0.279), followed by RWD (0.223), NHWD (0.123) and PERT (0.107). For Analysis 4—GWP D, Lower Env. Credit, RWD ranked highest at 0.223, while PENRT, PERT and reference unit had values of 0.188, 0.138, and 0.110, respectively. SM importance was 0.075 and 0.069, respectively. The importance of the EPD programme operator and country or region did not exceed 0.072. Higher PENRT, PERT, RWD, and NHWD values were associated with more negative predicted module D GWP, whereas the association for SM was weaker and less clear (Figure 6c,d).

PENRT mean SHAP was considerably lower in module D than in A1–A3 (0.188–0.279 against 0.513–0.524), whereas RWD and PERT had greater relative importance than in the A1–A3 models, as did SM relative to Analysis 1—GWP A1–A3, Base Dataset. These findings concern predictor importance within the models and should not be interpreted as evidence of causal relationships.

To complement the predictor-importance analysis, associations between quantitative A1–A3 indicators and module D GWP were assessed using Pearson and Spearman correlations. Table 6 presents the results.

PENRT showed the strongest association with module D GWP among the quantitative predictors. For Analysis 3—GWP D, Higher Env. Credit, the coefficients were Pearson r = −0.590 and Spearman ρ = −0.697. For Analysis 4—GWP D, Lower Env. Credit, the corresponding values were r = −0.575 and ρ = −0.625. PERT, RWD, and NHWD also showed negative correlations, with Spearman coefficients larger in absolute magnitude than their Pearson counterparts.

The linear association between SM and module D GWP was weak and not statistically significant in either variant: *r* = −0.041 for Analysis 3—GWP D, Higher Env. Credit and *r* = −0.025 for Analysis 4—GWP D, Lower Env. Credit. Spearman coefficients were *ρ* = −0.309 and *ρ* = −0.236, respectively. These correlations were calculated across the full population, which includes products declared using different reference units.

**Figure 7:**
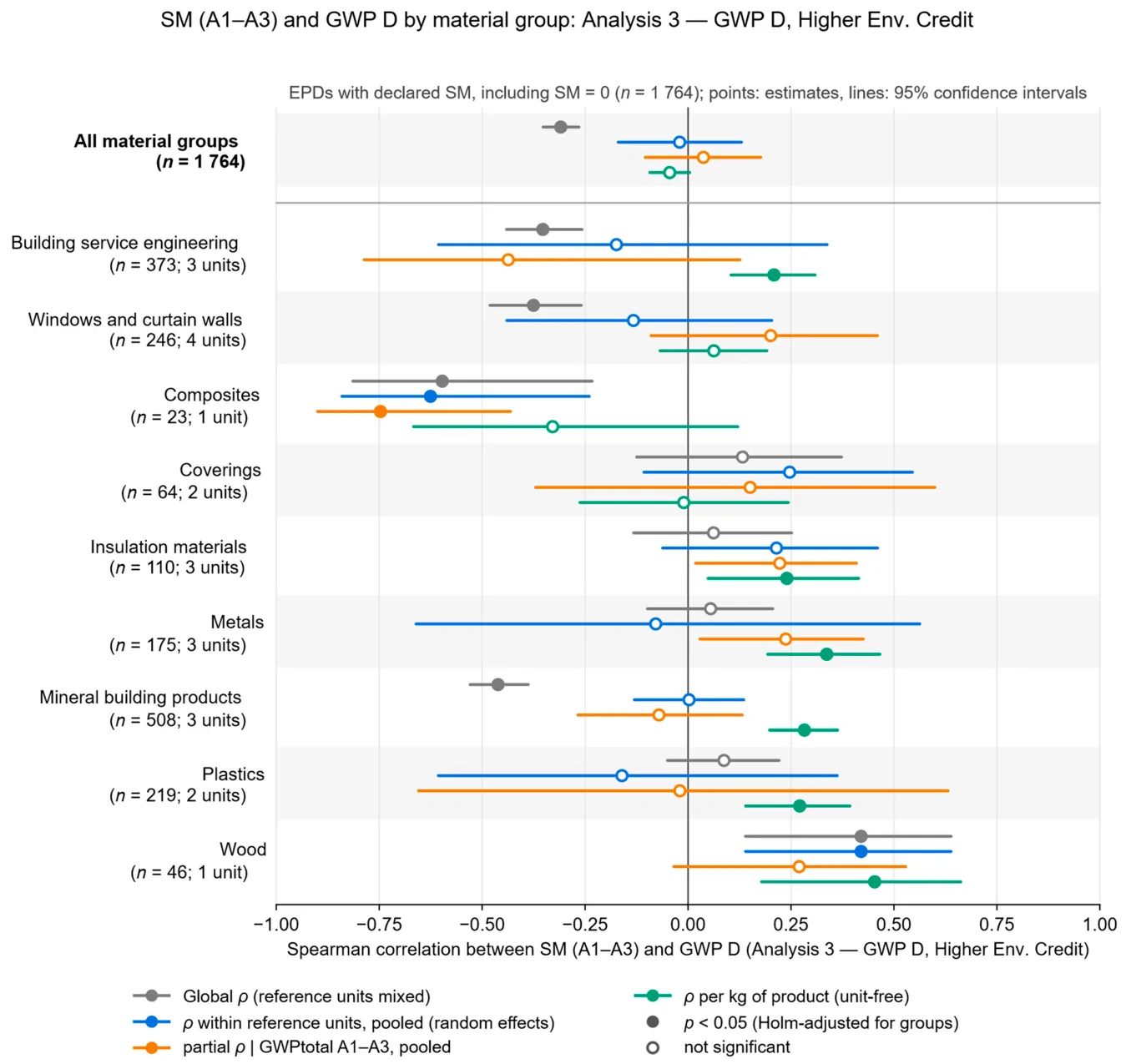
Spearman correlations between SM (A1–A3) and module D GWP (Analysis 3), for all material groups combined and for individual groups: overall (reference units pooled), within reference units (pooled random-effects estimate), partial correlations controlling for A1–A3 GWP–total (pooled), and correlations after conversion to a per-kilogram basis. Filled symbols: *p* < 0.05 after Holm correction; data in Table S21. Own elaboration.

Because SM and module D GWP are both declared per reference unit, the association between them was also examined within reference units and material groups (Figure 7; Table S21). In the full population (*n* = 1764), the overall Spearman coefficient was ρ = −0.309 (95% CI −0.352 to −0.265). When correlations were calculated within reference units and pooled, the association disappeared (ρ = −0.020; 95% CI −0.170 to 0.130); it also remained close to zero after adjustment for A1–A3 GWP–total (ρ = 0.037; −0.104 to 0.177) and after conversion to a per-kilogram basis (ρ = −0.045; −0.093 to 0.004). The same pattern occurred in the groups with significant overall correlations: building services (ρ = −0.353), window and curtain–wall components (ρ = −0.376), and mineral products (ρ = −0.461) showed within-unit coefficients of −0.174, −0.133, and 0.002, respectively, with confidence intervals including zero.

After conversion to 1 kg of product, Spearman correlations were positive and significant after Holm correction for metals (ρ = 0.337; 95% CI 0.194 to 0.466), mineral products (ρ = 0.282; 0.198 to 0.363), plastics (ρ = 0.271; 0.140 to 0.393), building services (ρ = 0.209; 0.104 to 0.308), wood (ρ = 0.453; 0.179 to 0.662), and, marginally, insulation materials (ρ = 0.240; 0.049 to 0.414; Holm-adjusted p = 0.048). A positive coefficient means that products with a higher SM mass fraction had higher, i.e., less negative, module D GWP values. After additional control for A1–A3 GWP–total per kg, only the correlation for mineral products remained significant after Holm correction (ρ = 0.283; 95% CI 0.199 to 0.364; *n* = 507; Table S21).

### 3.4. The Role of Secondary Material Use (SM) in GWP–Total Prediction

In the preceding analysis, SM had low relative mean |φ_j| SHAP in the Analysis 1 —GWP A1–A3, Base Dataset model (0.015) and moderate importance in the other datasets (0.069–0.075). Given the focus on secondary materials, the analysis examined SM in greater detail. Table 7 presents descriptive statistics for Analysis 1—GWP A1–A3, Base Dataset and Analysis 2—GWP A1–A3, SM > 0 Subset. In Analysis 1—GWP A1–A3, Base Dataset, 740 records reported SM = 0, and one record did not report SM. Among records with SM > 0, the median was 0.768 kg per reference unit, and the mean was 14.11 kg per reference unit. The first and third quartiles were 0.133 and 6.20 kg per reference unit, respectively, and the maximum was 1840.5 kg per reference unit. The large mean–median difference and high maximum indicate a strongly right-skewed SM distribution. These statistics combine values declared for different reference units; they do not describe average secondary material content in products. Table S2 reports the proportion of EPDs with SM > 0 and median SM by material group.

The low relative mean |φ_j| SHAP assigned to SM does not establish how much information it adds beyond the other predictors. The XGBoost ablation analyses assessed this contribution for all four datasets. Table 8 presents the results.

For Analysis 1, removing SM changed test R^2^ by +0.002 (95% CI −0.001 to 0.005), and for Analysis 2 by −0.009 (−0.022 to 0.003), corresponding to a 13.9% MSE increase. In module D, removing SM reduced test R^2^ from 0.791 to 0.760 in Analysis 3 (ΔR^2^ = −0.031; 95% CI −0.067 to 0.005) and from 0.814 to 0.781 in Analysis 4 (ΔR^2^ = −0.033; −0.071 to 0.002). Cross-validation R^2^ decreased from 0.725 to 0.698 and from 0.719 to 0.681, respectively. These changes corresponded to MSE increases of 14.8% and 17.6%. Because all four intervals included zero, the effects of removing SM were not statistically conclusive (Section 2.6). Removing the EPD programme operator changed test R^2^ by between −0.004 and 0.000, with all intervals including zero (Table 8).

### 3.5. Robustness Checks

Table 9 summarises the robustness analyses; details are given in Tables S15–S20. All outlier-handling variants were split before filtering and were therefore evaluated on two subsets of the same test set: all test records (*n* = 495, 326 and 445 for Analyses 1–3) and the test records within the published IQR range (*n* = 432, 294 and 363). With the IQR filter applied to the training set only, test R^2^ within the published range was 0.943, 0.977, and 0.660 for Analyses 1–3, compared with 0.963, 0.935, and 0.791 for the published models, whereas R^2^ on all test records was −0.006, −0.002, and −0.026. Selecting the module D scenario maximum before filtering (Analysis 4) gave the same pattern (0.632 within the range; −0.019 on all test records). Without outlier removal, R^2^ on all test records was 0.226, 0.458, and −2.617; a few extreme training records (e.g., 128,254 kg CO_2_eq. per chiller; Table S15) dominated model fitting, and R^2^ within the published range was negative. Not all extreme values were genuine: three aluminium profiles were declared at 13,181–41,671 kg CO_2_eq. per kg, about three orders of magnitude above standard profiles of the same manufacturer (8.15–8.33 kg CO_2_eq. per kg), although their PENRT was lower, which indicates errors in the source records (Table S15). With the asinh-transformed target, R^2^ within the published range was 0.935 and 0.963 for A1–A3 but −2.17 for module D, whereas on all test records, it was 0.053 and 0.192 for A1–A3 and 0.970 for module D (0.866 on the asinh scale). The median absolute relative error on all test records was 31.2% with the asinh target and 52.4% without transformation in Analysis 3, and 15.5% and 39.6% in Analysis 1. PENRT remained the first- or second-ranked SHAP predictor in all filtered or transformed variants, and the rank correlation with the published SHAP ranking was 0.53–0.95. The SM ablation effect remained small in A1–A3 (|ΔR^2^| ≤ 0.022) and negative in module D (−0.025 and −0.055) in the training-only filtering variants.

Repeated nested cross-validation gave mean outer-fold R^2^ values of 0.942, 0.952, and 0.755 for XGBoost and 0.934, 0.944, and 0.731 for Random Forest in Analyses 1–3, respectively (Table S19), close to the cross-validation results of the main models; the single-split test R^2^ of Analysis 3 (0.791) lay in the upper part of the outer-fold range (0.679–0.808). Under GroupKFold by declaration owner, the mean R^2^ of XGBoost decreased from 0.944 (random 5-fold cross-validation) to 0.871 in Analysis 1, from 0.951 to 0.893 in Analysis 2, and from 0.757 to 0.423 in Analysis 3; grouping by product category reduced it to 0.708, 0.793, and 0.392, respectively (Table S17). Random Forest showed the same pattern (module D: 0.496 and 0.419). In leave-one-operator-out validation, the pooled out-of-group R^2^ was 0.869, 0.786, and 0.340 (Table S16); records of the dominant operator (IBU) were predicted by models trained on the remaining operators with R^2^ = 0.926 in Analysis 1 and 0.294 in Analysis 3.

One-hot encoding of product category, or replacing it with the one-hot-encoded material group, changed the test R^2^ of Analysis 1 by −0.002 to +0.005 and increased that of Analysis 3 by 0.027–0.048, whereas cross-validation R^2^ of Analysis 3 changed by only −0.008 to +0.024 (Table S20). By dataset type, the test R^2^ of Analysis 1 was 0.968 for specific, 0.977 for average, 0.885 for generic, and 0.975 for template datasets; a model trained and tested on specific datasets only achieved R^2^ = 0.961 (Analysis 1) and 0.774 (Analysis 3). Signed errors were small relative to MAE for most reference units and material groups (Table S18, Figure S1). The clearest systematic deviations concerned wood products, which were overpredicted in all four analyses (median signed error 2.3–3.5 kg CO_2_eq. per reference unit; 70–89% of test records overpredicted; *n* = 9–11), insulation materials and plastics in Analysis 1 (72% and 84% of test records overpredicted), and metals in module D (predicted benefit too small for 62–74% of test records). The residual spread increased with the predicted magnitude (Figure S1).

## 4. Discussion

Prediction performance was substantially higher for A1–A3 than for module D, indicating that production-stage indicators capture production-related GWP more effectively than benefits and loads beyond the system boundary. The high A1–A3 prediction performance is consistent with previous EPD-based studies. Koyamparambath et al. [24] achieved R^2^ = 0.81 for GWP using Random Forest, while Neupane et al. [27] reported R^2^ values of approximately 0.88–0.90 after cleaning and harmonising EPD data. Against this background, the A1–A3 results, particularly for XGBoost and Random Forest, confirm the value of EPD data for GWP–total prediction. Direct comparisons of R^2^ nevertheless require caution because the studies differ in data coverage, predictor sets, and preprocessing. In both A1–A3 datasets, the tree-based models captured the relationships between the selected predictors and GWP well; their training R^2^ exceeded cross-validation scores, indicating some adaptation to the training data, but this did not translate into a clear deterioration in test performance. The XGBoost–Random Forest differences were, however, within bootstrap uncertainty in three of the four datasets (Table S8), so the two ensembles should be regarded as performing comparably.

A1–A3 information captured a substantial proportion of module D GWP variation, but module D prediction was more sensitive to sample composition: cross-validation R^2^ varied more between folds, test R^2^ exceeded cross-validation means, and the gap between training and cross-validation R^2^ indicated stronger overfitting than in A1–A3. The lower module D performance indicates that A1–A3 information captures only part of the GWP–total variation beyond the system boundary. This distinction matters because module D differs from the production stage. It addresses potential benefits and loads arising from activities such as reuse, material recovery, recycling, and energy recovery after a product reaches the end of its life. Its value is therefore closely tied to the declared end-of-life scenario and assumptions about subsequent material management. In their analysis of European EPDs, Anderson et al. [29] found substantial variation in how scenarios for modules C and D were defined and reported, hindering their direct use and comparison. Product-specific LCAs also show the importance of end-of-life assumptions. Cheong et al. [30] demonstrated that changing the end-of-life scenario for curtain–wall façades, particularly the shares of landfill and recycling, can substantially alter the assessment of environmental benefits when module D is included. Predicting module D may therefore require information not directly represented by the A1–A3 predictors. Relevant information concerns product management after use, including waste processing in C3, disposal in C4, recovery and recycling rates, and assumptions about materials or energy displaced beyond the system boundary.

The similarity between the module D results of Analyses 3 and 4 largely reflects the identical target values for 90.4% of EPDs and therefore should not be interpreted as evidence that scenario choice is unimportant. Because the two models were trained independently, their predictions also do not define an ordered interval (Table S9); Analysis 3 was therefore treated as the primary module D analysis and Analysis 4 as a scenario sensitivity check. The two variants also do not identify which end-of-life assumptions explain the remaining unexplained variation.

Group-aware validation sharpened the distinction between the two tasks. For A1–A3, R^2^ remained above 0.87 when entire manufacturers were held out and above 0.70 when entire product categories were held out, indicating that the A1–A3 relationships transfer reasonably well to unseen manufacturers. For module D, R^2^ fell from 0.76 under random cross-validation to 0.42 for unseen manufacturers and 0.39 for unseen product categories (Table 9). Lower performance under grouped validation indicates limited transfer beyond the declaration owners and product categories represented in training. It is consistent with a contribution of related records of the same manufacturer or category, such as product variants declared with a shared end-of-life scenario, but it does not by itself establish the extent of such near-duplicate leakage because the distribution of categories, manufacturers, and target values also differs between grouped folds. As the grouped folds used the hyperparameters tuned on the random split, they describe the transfer of a fixed model configuration. The module D results should be interpreted as interpolation within the database rather than as predictions for new manufacturers or product families. Applying the outlier filter to the training data only lowered module D R^2^ within the published range from 0.79 to 0.66, and on the complete test sets, which include records outside the training range, R^2^ was close to zero in all analyses (Table S15). The reported performance, particularly for module D, is therefore conditional on the outlier handling and applies only to products within the value range of the training data, which for a new product must be judged from its predictors. Extreme declared values, some of which are evident data errors, should be verified against the source EPD rather than removed or retained automatically. Relative predictor-importance patterns differed between the A1–A3 and module D models, indicating that the two prediction tasks rely on different environmental information. This is consistent with the distinction between the A1–A3 production stage and the benefits or loads reported beyond the system boundary in module D.

The low relative importance of programme operator in the A1–A3 models requires caution. The mean |φ_j| SHAP values (0.009–0.029) and ablation results indicate that this variable added little useful information beyond the quantitative indicators. They do not, however, support a causal interpretation. Earlier ML studies assigned considerable importance to the EPD programme operator, interpreting it as a possible indicator of heterogeneity between EPD programmes [27]. Differences in data composition may explain this contrast. Although the dataset used covered seven operators, one operator registered 61% of the A1–A3 EPDs (IBU). The earlier studies used ECO Portal data with a more diverse distribution of operators. Despite the shared EN 15804+A2 framework, EPD programmes can differ in their General Programme Instructions, Program Category Rules (PCR), and detailed modelling rules [8]. However, the operator variable added no predictive information beyond the other predictors in our dataset. It is only a proxy for these differences, without direct information on PCR, allocation rules, or background data. The choice of interpretation measure also matters: XGBoost gain assigned high importance to categorical variables, with large differences across datasets (operator: 0.06–0.43; country or region: 0.07–0.47; Table S12). SHAP did not corroborate these patterns, nor did ablation for the operator variable. These results support further investigation of programme-operator effects in databases covering multiple operators. Leave-one-operator-out validation complements this picture: models trained without the dominant operator (IBU) still predicted its A1–A3 records with R^2^ = 0.926, whereas performance for small operators (Bau EPD, Dachverband Lehm; *n* ≤ 39) was poor (Table S16). The operator variable itself therefore added little predictive information. In this validation, the held-out operator is absent from training by design, and the poor results for operators with few records (*n* ≤ 39) show that transfer to programme operators not included in the training data is uncertain.

PENRT warrants particular attention among the quantitative environmental indicators. It showed a strong monotonic association with A1–A3 GWP–total (ρ = 0.892; r = 0.532) and was the most important XGBoost predictor. This has an environmental basis: PENRT represents total use of non-renewable primary energy resources, and the processes consuming these resources often also generate greenhouse gas emissions, as reported in [24]. The relationship is not uniform across material groups, however. In an analysis of structural timber EPDs, Rasmussen et al. found only a weak correlation between A1–A3 GWP and PENRT (R^2^ = 0.1162), and an even weaker correlation between A1–A3 GWP and PERT (R^2^ = 0.0192) [41]. The high predictive value of PENRT in the present study should therefore not be treated as universal across construction products. It may partly reflect the broad and heterogeneous product coverage of the dataset.

The comparison between PENRT and PERT is informative. PERT also showed a clear monotonic association with GWP–total (ρ = 0.694; r = 0.066), yet its relative mean |φ_j| SHAP in the A1–A3 models was much lower (0.042–0.043). High PERT values were associated with negative SHAP values, potentially reflecting products made from renewable resources such as wood. This interpretation remains a hypothesis to be tested within material groups. A strong correlation between one predictor and the target therefore need not translate directly into high importance in a multivariable model. Environmental indicators may contain overlapping information, and the model uses their combinations rather than each predictor independently. This also matters when interpreting SM.

Module D models showed a different pattern. Alongside PENRT, RWD, NHWD, and PERT had high relative importance, and higher values of these indicators were associated with higher environmental benefits in module D GWP–total. RWD may be influenced by radioactive waste associated with nuclear electricity generation and may therefore be related to the share of nuclear power in the electricity mix [42]. Its mean |φ_j| SHAP alone cannot reveal whether the model uses information about the regional energy mix, waste management, recycling practices, product composition, or other location-associated characteristics. Similarly, PENRT and PERT may partly represent differences between product types and reporting practices, such as greater potential recycling benefits for metal products. Country or region and programme operator had low importance in the module D models.

SM requires particular consideration because secondary material use is a central focus of this study. In the A1–A3 models, SM provided limited additional predictive information beyond the other environmental indicators and EPD metadata. One possible explanation is partial redundancy, as PENRT, PERT, and other indicators may already capture characteristics of materials and production processes indirectly. The positive SHAP contribution of SM should therefore not be interpreted as evidence that secondary material use increases GWP. SM played a clearer role in module D, although the ablation results were not statistically conclusive. The material-group analysis shows that the negative overall correlation between SM and module D GWP does not imply that greater use of secondary materials increases the module D benefit. The association arose from pooling products of different sizes was declared using different reference units. On a per-kilogram basis, higher SM mass fractions were associated with smaller module D benefits in several groups, including metals, plastics, and mineral products. This direction is consistent with the EN 15804 principle that module D benefits are assigned to the net material flow: the quantity recovered at the end of the life cycle minus the secondary material used as input. The correlations were moderate, however, and weakened in most groups after adjustment for A1–A3 GWP–total. They therefore cannot be taken as evidence of a causal relationship. This is consistent with the calculation of module D, which depends not only on secondary material input but also on recovered quantities, recovery and recycling efficiencies, secondary material quality, and the production displaced. SM therefore represents only one component of the secondary material flow balance and cannot determine the module D result on its own. The potential benefits reported in Module D arise from avoided primary production enabled by the use of recovered material. However, the displaced primary material does not necessarily have to correspond to the same product or serve the same application as the original product from which the material was recovered. For example, concrete demolition waste may be processed into recycled aggregate and used in applications where it substitutes natural aggregate rather than the original concrete product [43]. This depends on the recycling route, the quality of the secondary material, and its intended end use.

The per-kilogram result also has a practical implication. Under the net-flow approach of EN 15804+A2, secondary material used as input reduces the net output flow that can be credited in module D. A higher declared SM should therefore not be expected to translate into a larger module D credit; at equal recovered quantities, it reduces the credit. Comparing products on module D alone may thus disadvantage products with high secondary material content, whose benefit is already reflected in lower A1–A3 burdens, so module D should be interpreted together with A1–A3, as EN 15804+A2 requires its separate reporting. For predictive modelling, SM is informative about module D only in combination with information on end-of-life recovery; an indicator of the net flow, i.e., the quantity of materials for recycling at the end of life minus the secondary material input, would be a more appropriate predictor. The per-kilogram analysis was exploratory: it relied on mass data available for 1741 EPDs, sample sizes were small for several groups, and after control for A1–A3 GWP–total, only the association for mineral products remained statistically significant (Figure 7).

The performance difference between A1–A3 and module D suggests that the use of ML to complete missing EPD data should depend on the indicator being predicted. For A1–A3 GWP, the results suggest that models could support estimation of missing values when the required predictors are available. However, performance was assessed on complete records. Records missing GWP–total may differ in publication period, operator, or standard version, so imputation requires separate validation in that population. Greater caution is needed for module D: the current variables explained approximately 72–81% of its variation, and prediction quality depended more strongly on sample composition. These models could serve as supplementary or screening tools rather than replace information derived directly from a declared end-of-life scenario.

Based on these results, the domain of validity and recommended use of the models can be specified. They should be applied only (i) to EN 15804+A2 records from product categories, reference units, and value ranges represented in the training data (here, A1–A3 GWP–total from −129 to 222 kg CO_2_eq. and module D GWP–total from −24 to 5 kg CO_2_eq. per reference unit); (ii) as screening estimates for comparing material or design variants, or for plausibility checks of declared values, not as substitutes for verified EPD results; and (iii) with error margins reported per reference unit rather than as a single global value. The typical relative error was about 12–13% of the mean absolute A1–A3 GWP–total and about 27% for module D, but it was larger for products declared per kg and for low-GWP products, for which the log–log baseline achieved better relative accuracy (Tables S5 and S7). Records outside the training range, from categories absent from training, or from manufacturers and operators not represented in the data should be flagged because performance declined in these cases (Tables S6, S16 and S17). The systematic deviations identified for wood, insulation materials, and metals (Table S18) indicate groups that require group-specific models or additional predictors. Formal prediction intervals were not derived in this study; their development, for example, by conformal prediction stratified by reference unit, is a natural next step before operational use.

The high A1–A3 prediction performance must also be interpreted in light of the inputs. Many quantitative predictors, including PENRT, PERT, RWD, and NHWD, are environmental indicators calculated within the same LCA model as GWP–total. High *R*^2^ may therefore partly reflect shared information about the processes and flows underlying these indicators. This is not direct target leakage, but it limits interpretation of the results as GWP prediction from simple product properties alone. In practice, using such a model to estimate missing values would require at least some of the other environmental indicators.

Because the dataset spans diverse product categories and reference units, aggregate performance does not imply equally accurate predictions within every material group. Further validation should assess individual product categories to establish whether the observed relationships are general or partly reflect the structure of the heterogeneous dataset. Tables S14 and S18 provide results by material group. The analysis of the relationship between SM and module D GWP also showed that the direction of an association inferred from the full population can change once reference unit and material group are taken into account.

Several limitations affect the interpretation of these findings. First, target-based IQR filtering preceded the train–test split and removed records unevenly. In A1–A3, exclusions included 27.0% of records declared per m^3^ and 57.5% of timber products. In module D, they included 45.7% of window and curtain–wall components and all module D records for 462 EPDs (Table S4). The results therefore describe the filtered population. Applying the filter to the training data only changed A1–A3 performance within the published range little (*R*^2^ = 0.943 and 0.977) but reduced module D *R*^2^ from 0.79 to 0.66, and on all test records, including those outside the training range, *R*^2^ was close to zero in all analyses (Table S15). Second, the dataset combines values declared for different reference units. Global MAE and RMSE therefore lack a uniform practical interpretation, and performance varied across units (Table S5). Third, the main results rely on a single random split, which does not rule out dependence between related records, such as product variants from one manufacturer. Removing 133 identical quantitative profiles (Table S3) reduces but does not eliminate this problem; grouped validation showed lower performance for unseen manufacturers and categories, particularly for module D (Tables S16 and S17). Fourth, 56 of the 221 categories contained a single record, and frequency encoding does not preserve category identity (Table S10). This limits assessment of generalisation to new products, particularly those in categories absent from training (Table S6). Fifth, the module D targets of Analyses 3 and 4 were identical for 90.4% of EPDs. Finally, ablation held hyperparameters fixed, and the uncertainty intervals for performance changes after removing SM included zero (Table S8). The per-kilogram analysis of SM and module D GWP relied on physical properties reported in ÖKOBAUDAT, which may be incomplete or inconsistent in some cases. Sample sizes were also small for several material groups, notably wood, composites, and coverings. Future research should validate the models over time, using later ÖKOBAUDAT releases, and develop prediction intervals. For module D, richer end-of-life information and joint modelling of alternative scenarios could improve prediction and uncertainty assessment. Validation using later ÖKOBAUDAT releases, other EPD databases, and more detailed information on secondary materials would help assess the generalisability of the identified relationships. Another relevant direction is to assess whether GWP–total can be predicted from more readily available product-level information, such as declared material composition, secondary material content, and basic technical or metadata characteristics. This could reduce dependence on other environmental indicators derived from LCA and support earlier environmental screening before a complete LCA or full EPD indicator set is available. Further analyses should also include the quantity of material sent for recycling at the end of the life cycle. This would allow an assessment of whether the net flow of secondary material explains module D GWP better than SM alone. The modelling framework could also be extended beyond GWP–total to other environmental impact indicators reported in EPDs, allowing assessment of whether the observed predictor relationships and model performance are specific to GWP or generalise across impact categories.

## 5. Conclusions

- Machine-learning models predicted A1–A3 GWP–total accurately in the analyzed dataset. XGBoost achieved the highest point estimates, with R^2^ = 0.963 for Analysis 1 and R^2^ = 0.935 for Analysis 2. Random Forest produced similar results, and the difference between the two was within bootstrap uncertainty in three of the four datasets, whereas SVR performed less well. These results refer to records retained after IQR-based removal of target outliers; with outliers removed from the training data only, A1–A3 performance was similar (R^2^ = 0.94–0.98), but the values outside the training range were not reproduced (R^2^ ≈ 0 on all test records).
- Module D GWP was considerably harder to predict than A1–A3 GWP. XGBoost achieved R^2^ = 0.791 for Analysis 3, the primary module D analysis; the scenario variant (Analysis 4, R^2^ = 0.814) gave a similar result because its targets were identical for 90% of EPDs. Production-stage information therefore explains a substantial, but incomplete, proportion of the variation in module D results within the database, and generalization to unseen manufacturers and product categories was limited (R^2^ ≈ 0.4).
- Predictor-importance patterns differed between A1–A3 and module D. PENRT dominated the A1–A3 models, whereas RWD, PERT, and SM gained relative importance in module D as PENRT importance declined.
- SM provided limited additional information for A1–A3 GWP–total prediction, but its role was clearer in module D. Removing SM from module D models reduced R^2^ by approximately 0.03 and increased MSE by approximately 15–18%. However, the bootstrap intervals included zero, so the effect was not statistically conclusive.
- SM should not be interpreted as a standalone indicator of recycling potential or a direct determinant of module D results. It is one component of the secondary material flow balance. Module D also depends on the quantity of recovered material, recovery and recycling efficiencies, secondary material quality, and the type of production displaced.
- ML models can support the estimation of module D GWP–total. Once the A1–A3 indicators of an EPD are available, the models can be used to estimate a missing module D value and to check the plausibility of declared A1–A3 and module D values, provided that the product lies within the domain of the training data. Such estimates should support, not replace, an assessment based on a declared end-of-life scenario, particularly for manufacturers and product categories that are not represented in the training data.
- Further research should extend the module D predictor set to include information on C3–C4, recovery and recycling rates, and detailed end-of-life assumptions. Models also require validation within individual material groups and on independent EPD datasets.

## Figures and Tables

**Table 1:** Characteristics of the analytical datasets and target distributions after data cleaning. Own elaboration.

Dataset	Target Module	*n*	*n* Training/Test	Median	Q1–Q3	Min–Max	Negative GWP–Total Values [%]
Analysis 1—GWP A1–A3, Base Dataset	A1–A3	2214	1771/443	8.06	1.58–47.47	−129.38–221.66	4.4
Analysis 2—GWP A1–A3, SM > 0 Subset	A1–A3	1473	1178/295	11.55	2.49–83.04	−72.72–220.00	3.5
Analysis 3—GWP D, Higher Env. Credit	D (min.)	1765	1412/353	−1.71	−7.31–−0.36	−23.98–5.16	93.1
Analysis 4—GWP D, Lower Env. Credit	D (max.)	1765	1412/353	−1.46	−6.76–−0.17	−23.98–5.16	86.9

Target values are expressed in kg CO_2_eq. per reference unit. The train–test split was 80/20.

**Table 2:** Target and predictors used in the machine-learning models. Own elaboration.

Group	Variable	Description	Type	Encoding
Target	GWP–total	Global Warming Potential total of A1–A3 or module D [kg CO_2_eq./RU]	Quantitative	–
Quantitative predictors (A1–A3)	PENRT	Total use of non-renewable primary energy resources [MJ/RU]	Quantitative	None *
	PERT	Total use of renewable primary energy resources [MJ/RU]	Quantitative	None *
	SM	Use of secondary material [kg/RU]	Quantitative	None *
	RWD	Radioactive waste disposed [kg/RU]	Quantitative	None *
	NHWD	Non-hazardous waste disposed [kg/RU]	Quantitative	None *
Categorical predictors (metadata)	Product category	Hierarchical ÖKOBAUDAT product classification (221 values)	Categorical	Frequency
	Country/region	ISO 3166-1 [33] country code or regional code (34 values)	Categorical	one-hot
	Reference unit	m^2^, kg, piece, m^3^, m, MJ (6 values)	Categorical	one-hot
	EPD programme operator	Organisation that registers and publishes EPDs (7 values after harmonisation)	Categorical	one-hot

* Values were used without transformation. For SVR, all encoded predictors were standardised (mean 0, standard deviation 1) numbers of categorical levels refer to Analysis 1—GWP A1–A3, Base Dataset.

**Table 3:** Hyperparameter search spaces and selected values for each model and dataset. Own elaboration.

Model	Hyperparameter	Search Space	Analysis 1—GWP A1–A3, Base Dataset	Analysis 2—GWP A1–A3, SM > 0 Subset	Analysis 3—GWP D, Higher Env. Credit	Analysis 4—GWP D, Lower Env. Credit
XGBoost	n_estimators	200, 300, 500, 800	800	300	300	800
	learning_rate	0.01; 0.03; 0.05; 0.1; 0.2	0.01	0.1	0.05	0.1
	max_depth	3, 4, 5, 6, 8, 10	10	6	10	5
	min_child_weight	1, 3, 5, 10	5	3	3	5
	subsample	0.6–1.0 (by 0.1)	0.8	0.6	0.7	0.6
	colsample_bytree	0.5–1.0 (by 0.1)	0.8	0.6	1	0.7
	reg_lambda	0.1; 1; 5; 10	10	1	10	10
	reg_alpha	0;0.01; 0.1; 1	0.1	0.1	0	1
Random Forest	n_estimators	200, 300, 500	500	300	300	300
	max_depth	None, 10, 20, 30	30	30	None	None
	min_samples_split	2, 5, 10	2	2	2	2
	min_samples_leaf	1, 2, 4	2	1	1	1
	max_features	1.0; 0.5; 0.33; sqrt	1	1	0.33	0.33
SVR (RBF)	C	0.1; 1; 10; 100; 1000	100	10	10	10
	ε	0.01; 0.05; 0.1; 0.2; 0.5	0.01	0.01	0.2	0.2
	γ	scale; 0.001; 0.01; 0.1	0.01	scale	0.1	0.1

Initial configuration: XGBoost–n_estimators = 300, learning_rate = 0.05, max_depth = 6, subsample = 0.8, colsample_bytree = 0.8; RF–n_estimators = 200, max_depth = None; SVR–C = 10, ε = 0.1, γ = scale. Parameters not listed used the library defaults. For SVR, ε refers to the standardised target.

**Table 4:** Predictive model performance for Analysis 1—GWP A1–A3, Base Dataset and Analysis 2—GWP A1–A3, SM > 0 Subset. Own elaboration.

Dataset	Model	Training R^2^	CV R^2^ (Mean ± SD)	Test R^2^	Test MAE *	Test RMSE *
Analysis 1—GWP A1–A3, Base Dataset	XGBoost	0.990	0.937 ± 0.020	0.963	5.30	11.98
	Random Forest	0.985	0.935 ± 0.022	0.958	5.52	12.73
	SVR (RBF)	0.965	0.907 ± 0.037	0.941	5.61	15.13
Analysis 2—GWP A1–A3, SM > 0 Subset	XGBoost	0.999	0.964 ± 0.007	0.935	6.29	17.04
	Random Forest	0.994	0.954 ± 0.011	0.915	6.76	19.48
	SVR (RBF)	0.985	0.939 ± 0.037	0.887	7.77	22.46

* MAE and RMSE are expressed in kg CO_2_eq. per reference unit of the respective EPD record.

**Table 5:** Predictive model performance for Analysis 3 and Analysis 4. Own elaboration.

Dataset	Model	Training R^2^	CV R^2^ (Mean ± SD)	Test R^2^	Test MAE *	Test RMSE *
Analysis 3—GWP D, Higher Env. Credit	XGBoost	0.990	0.725 ± 0.072	0.791	1.14	2.27
	Random Forest	0.961	0.706 ± 0.062	0.775	1.22	2.35
	SVR (RBF)	0.796	0.588 ± 0.035	0.680	1.57	2.81
Analysis 4—GWP D, Lower Env. Credit	XGBoost	0.995	0.719 ± 0.084	0.814	1.18	2.13
	Random Forest	0.960	0.692 ± 0.073	0.790	1.18	2.26
	SVR (RBF)	0.792	0.586 ± 0.029	0.665	1.59	2.85

* MAE and RMSE are expressed in kg CO_2_eq. per reference unit of the respective EPD record.

**Table 6:** Pearson (*r*) and Spearman (*ρ*) correlations between A1–A3 predictors and module D GWP. Own elaboration.

Predictor	r (Analysis 3—GWP D, Higher Env. Credit)	ρ (Analysis 3—GWP D, Higher Env. Credit)	r (Analysis 4—GWP D, Lower Env. Credit	ρ (Analysis 4—GWP D, Lower Env. Credit)
SM	−0.041 *	−0.309	−0.025 *	−0.236
PENRT	−0.590	−0.697	−0.575	−0.625
PERT	−0.342	−0.619	−0.332	−0.545
RWD	−0.281	−0.675	−0.288	−0.624
NHWD	−0.289	−0.628	−0.253	−0.597

* *p* = 0.089 (Analysis 3—GWP D, Higher Env. Credit) and *p* = 0.29 (Analysis 4—GWP D, Lower Env. Credit)—correlations not statistically significant; all other correlations: *p* < 0.001; *n* = 1765 (the missing SM value was replaced with zero; results for complete pairs of observation–Table S13).

**Table 7:** Descriptive statistics for secondary material use (SM) in the A1–A3 datasets. Own elaboration.

Statistic	Analysis 1—GWP A1–A3, Base Dataset (*n* = 2214)	Analysis 2—GWP A1–A3, SM > 0 Subset (*n* = 1473)
*n* (SM = 0)	740	0
*n* (SM missing)	1	0
*n* (SM > 0)	1473	1473
Positive values (%)	66.5%	100%
Median (SM > 0)	0.768	0.768
Mean (SM > 0)	14.11	14.11
Q1 (SM > 0)	0.133	0.133
Q3 (SM > 0)	6.20	6.20
Maximum	1840.5	1840.5

**Table 8:** XGBoost ablation results (fixed hyperparameter configuration) with paired bootstrap 95% confidence intervals. Own elaboration.

Dataset	Variant	Test R^2^	CV R^2^	ΔR^2^ Test [95% CI]	ΔMSE [%] [95% CI]
Analysis 1—GWP A1–A3, Base Dataset	base	0.9627	0.9371	–	–
Analysis 1—GWP A1–A3, Base Dataset	without SM	0.9643	0.9391	+0.0016 [−0.0013, 0.0046]	−4.2 [−10.3, 4.1]
Analysis 1—GWP A1–A3, Base Dataset	without operator	0.9628	0.9381	0.0001 [−0.0021, 0.0019]	−0.2 [−5.4, 5.1]
Analysis 2—GWP A1–A3, SM > 0 Subset	base	0.9349	0.9644	–	–
Analysis 2—GWP A1–A3, SM > 0 Subset	without SM	0.9259	0.9621	−0.0091 [−0.0215, 0.0025]	+13.9 [−4.7, 35.8]
Analysis 2—GWP A1–A3, SM > 0 Subset	without operator	0.9318	0.9599	−0.0032 [−0.0137, 0.0082]	+4.9 [−13.0, 26.9]
Analysis 3—GWP D, Higher Env. Credit	base	0.7906	0.7248	–	–
Analysis 3—GWP D, Higher Env. Credit	without SM	0.7597	0.6981	−0.0309 [−0.0665, 0.0049]	+14.8 [−2.4, 34.4]
Analysis 3—GWP D, Higher Env. Credit	without operator	0.7867	0.7214	−0.0039 [−0.0228, 0.0128]	+1.9 [−6.6, 11.0]
Analysis 4—GWP D, Lower Env. Credit	base	0.8136	0.7194	–	–
Analysis 4—GWP D, Lower Env. Credit	without SM	0.7808	0.6807	−0.0328 [−0.0711, 0.0015]	+17.6 [−0.8, 38.4]
Analysis 4—GWP D, Lower Env. Credit	without operator	0.8129	0.7090	−0.0008 [−0.0166, 0.0142]	+0.4 [−7.4, 9.6]

**Table 9:** Summary of robustness checks (XGBoost unless stated otherwise). Own elaboration.

Robustness Check	Metric [Unit]	Analysis 1	Analysis 2	Analysis 3	Details
Published models: single split, IQR filter before split	test R^2^ [–]	0.963	0.935	0.791	Table 4 and Table 5
Relative MAE (MAE ratio to baseline)	relative MAE [%] (MAE ratio [–])	12.6 (0.33)	11.9 (0.39)	26.8 (0.53)	Table S5
IQR filter on training set only, global	test R^2^, all test records/within published range [–]	−0.006/0.943	−0.002/0.977	−0.026/0.660	Table S15
IQR filter on training set only, per reference unit	test R^2^, all test records/within published range [–]	−0.005/0.900	−0.003/0.922	−0.009/0.296	Table S15
No outlier removal	test R^2^, all test records/within published range [–]	0.226/−4.85	0.458/−28.05	−2.62/−2194.7	Table S15
No outlier removal, asinh-transformed target	test R^2^, all test records/within published range [–]	0.053/0.935	0.192/0.963	0.970/−2.17	Table S15
Repeated nested CV (5 folds × 3 repeats), XGBoost	outer-fold R^2^, mean (SD) [–]	0.942 (0.024)	0.952 (0.011)	0.755 (0.041)	Table S19
Repeated nested CV, Random Forest	outer-fold R^2^, mean (SD) [–]	0.934 (0.022)	0.944 (0.013)	0.731 (0.033)	Table S19
Random 5-fold CV, all records	CV R^2^, mean [–]	0.944	0.951	0.757	Table S17
GroupKFold by declaration owner (manufacturer)	CV R^2^, mean [–]	0.871	0.893	0.423	Table S17
GroupKFold by product category	CV R^2^, mean [–]	0.708	0.793	0.392	Table S17
Leave-one-operator-out	pooled out-of-group R^2^ [–]	0.869	0.786	0.340	Table S16
One-hot product category instead of frequency encoding	test R^2^ [–]	0.961	–	0.818	Table S20
Material group (one-hot) instead of product category	test R^2^ [–]	0.966	–	0.837	Table S20
Specific datasets only (training and test)	test R^2^ [–]	0.961	–	0.774	Table S20

Rows 3–6: data were split before outlier handling; the first value refers to all test records (*n* = 495, 326 and 445), the second to the test records within the published IQR interval (*n* = 432, 294 and 363). The published interval is used only to partition the test set; it cannot be checked for a new product whose GWP–total is unknown. a–*R*^2^ on the asinh scale, all test records. Rows 9–12: tuned hyperparameters of the main models; Random Forest results in Table S17. Relative MAE–MAE divided by the mean absolute observed value; in brackets, MAE ratio to the category × unit median baseline. R^2^ and the MAE ratio are dimensionless [–]. – not analysed.

## Data Availability

The original contributions presented in this study are included in the article/Supplementary Material. Further inquiries can be directed to the corresponding author.

## Supplementary Materials

The following supporting information can be downloaded at: https://www.mdpi.com/article/10.3390/ma19214666/s1, Table S1: Dataset types in Analysis 1 (n = 2214); Table S2: Material-group characteristics and module D coverage; Table S3: Audit of 133 duplicate A1–A3 quantitative profiles; Table S4: IQR exclusions by reference unit and material group; Table S5: Test performance by reference unit; Table S6: Test performance for seen and unseen product categories; Table S7. Comparison with baseline models; Table S8: Bootstrap confidence intervals for model performance differences; Table S9: Module D scenario variability and prediction performance; Table S10: Frequency-encoding collisions for product categories; Table S11: Sensitivity of SHAP importance to aggregation methods; Table S12: Comparison of XGBoost gain and SHAP importance; Table S13: Complete-case correlations between A1–A3 predictors and module D GWP; Table S14: XGBoost test performance by material group; Table S15: Sensitivity of XGBoost predictions to outlier handling; Table S16: Leave-one-programme-operator-out validation; Table S17: Random versus group-based cross-validation; Table S18: Signed prediction errors by reference unit and material group; Table S19: Repeated nested cross-validation of XGBoost and Random Forest; Table S20: Effects of dataset type and category encoding on XGBoost performance; Table S21: SM–module D GWP correlations by material group and reference unit; Figure S1: XGBoost residuals versus predicted GWP by reference unit (Analyses 1–4).

## Abbreviations

The following abbreviations are used in this manuscript:

A1–A3	Product stage modules: raw material supply (A1), transport to the manufacturer (A2), manufacturing (A3)
C3	End-of-life module: waste processing
C4	End-of-life module: disposal
CI	Confidence interval
CO_2_eq.	Carbon dioxide equivalent
CRU	Components for re-use
CV	Cross-validation
D	Module D: benefits and loads beyond the system boundary (reuse, recovery and recycling potential)
EEE	Exported electrical energy
EET	Exported thermal energy
EN	European Standard
Env.	Environmental
EPD	Environmental Product Declaration
FW	Net use of fresh water
GLO	Global (regional code)
GWP	Global Warming Potential
GWP–biogenic	Global Warming Potential–biogenic
GWP–fossil	Global Warming Potential–fossil fuels
GWP–luluc	Global Warming Potential–land use and land use change
GWP–total	Global Warming Potential–total, reported according to EN 15804+A2
HWD	Hazardous waste disposed
IBU	Institut Bauen und Umwelt e.V.
IQR	Interquartile range
ISO	International Organization for Standardization
LCA	Life-Cycle Assessment
MAE	Mean Absolute Error
MER	Materials for energy recovery
MFR	Materials for recycling
ML	Machine Learning
MSE	Mean Squared Error
NHWD	Non-hazardous waste disposed
NRSF	Use of non-renewable secondary fuels
PCR	Product Category Rules
PENRE	Use of non-renewable primary energy excluding non-renewable primary energy resources used as raw materials
PENRM	Use of non-renewable primary energy resources used as raw materials
PENRT	Total use of non-renewable primary energy resources
PERE	Use of renewable primary energy excluding renewable primary energy resources used as raw materials
PERM	Use of renewable primary energy resources used as raw materials
PERT	Total use of renewable primary energy resources
Q1, Q3	First and third quartile
R^2^	Coefficient of determination
RBF	Radial Basis Function
RER	Europe (regional code)
RF	Random Forest
RMSE	Root Mean Squared Error
RNA	North America (regional code)
RSF	Use of renewable secondary fuels
RU	Reference unit
RWD	Radioactive waste disposed
SD	Standard deviation
SHAP	SHapley Additive exPlanations
SM	Use of secondary material
SVR	Support Vector Regression
UUID	Universally Unique Identifier
WEU	Western Europe (regional code)
XGBoost	Extreme Gradient Boosting
